# Persistent Green Luminescence in Nanoparticles Functionalized with SARS-CoV‑2 Spike Proteins: Virus-Like Particles Showing Active Targeting toward Selected Cells

**DOI:** 10.1021/acsabm.5c01355

**Published:** 2025-10-26

**Authors:** Piotr Kuich, Urszula Bazylińska, Julita Kulbacka, Vitalii Boiko, Dariusz Hreniak, Michał Jewgiński, Marcin Nyk, Dominika Wawrzyńczyk

**Affiliations:** † Institute of Advanced Materials, Faculty of Chemistry, Wroclaw University of Science and Technology, Wybrzeze Wyspianskiego 27, 50-370 Wroclaw, Poland; ‡ Department of Physical and Quantum Chemistry, Faculty of Chemistry, Wroclaw University of Science and Technology, Wybrzeze Wyspianskiego 27, 50-370 Wroclaw, Poland; § Department of Molecular and Cellular Biology, Faculty of Pharmacy, 49550 Wroclaw Medical University, Borowska 211 A, 50-556 Wroclaw, Poland; ∥ Department of Immunology and Bioelectrochemistry, State Research Institute Centre for Innovative Medicine, LT-08406 Vilnius, Lithuania; ⊥ Division of Optical Spectroscopy, Institute of Low Temperature and Structure Research, Polish Academy of Sciences, Okolna 2, 50-422 Wroclaw, Poland; # Department of Physics of Biological Systems, Institute of Physics, National Academy of Sciences of Ukraine, Prospekt Nauky 46, UA-03028 Kyiv, Ukraine; ¶ Department of Bioorganic Chemistry, Faculty of Chemistry, Wroclaw University of Science and Technology, Wybrzeze Wyspianskiego 27, 50-370, Wroclaw, Poland

**Keywords:** persistent luminescence, nanoparticle surface functionalization, smart nanomaterials, SARS-CoV-2 S1 spike protein, enhanced cellular uptake

## Abstract

The possibility of using nanoparticles (NPs) showing prolonged luminescence in the biosensing and bioimaging fields of science is now attracting increasing attention. Such materials can help to overcome the problems of autofluorescence or increased photodamage because, for example, fractionated irradiance can be used in the light-induced generation of reactive oxygen species experiments. However, the carefully designed and engineered surface functionalization of those NPs is required for not only decreased toxicity but most importantly for active targeting toward selected cell types. As a solution, herein, we propose the construction of virus-like particles, which by mimicking the properties of real viruses can selectively enter cells with Toll-like receptors (TLRs), and additionally show afterglow emissive properties for enhanced biosensing and bioimaging applications. In particular, we have functionalized the surface of rod-like, Mn-doped, Zn_2_GeO_4_ NPs, showing efficient green persistent luminescence, with so-called “artificial corona” composed of SARS-CoV-2 S1 spike proteins. The designed material preserved unique optical properties, including stable green luminescence with persistent decay time at the level of several dozen seconds, showed decreased cytotoxicity, and, most importantly, was taken up more readily by the human pancreatic cancer cell lines positive for TLRs with respect to the cell line negative for those markers.

## Graphical abstract

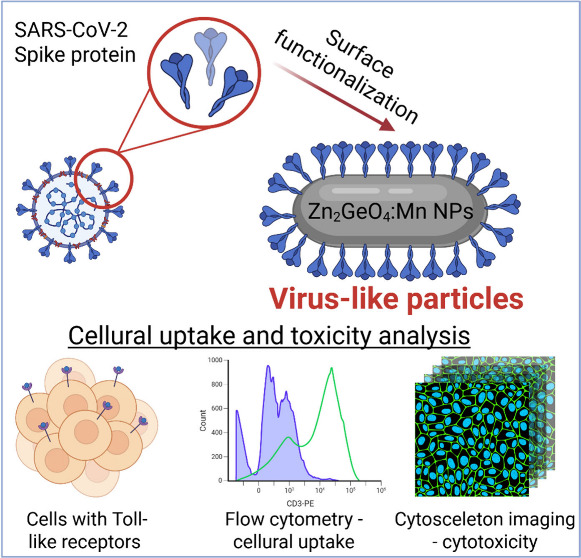

## 1 Introduction

The intriguing optical properties of persistent luminescent NPs (PersLNPs) make them interesting candidates for bioimaging and biosensing applications.^1–3^ These materials are characterized by the ultralong afterglow that can last after the excitation source is stopped.^4^ In such a condition, the problem of background autofluorescence and light scattering can be easily overcome due to the difference in characteristic emission kinetics; i.e., the images of emission from PersLNPs can be captured after the short component of background autofluorescence has decayed.^5–7^ However, the biodistribution, cytotoxicity, and metabolism of any type of NPs strongly depend on their surface state, i.e., charge, presence of biologically active molecules, or targeting factors.^8,9^ Therefore, engineered surface functionalization of PersLNPs for specific biorelated applications is of great importance and can open new perspectives for these types of materials. To date, several studies have shown efficient ways to introduce functional organic molecules onto the surface of PersLNPs to improve their stability in aqueous solutions and further enhance their performance in biorelated fields.^10^ In the paper published by Wang et al.,^11^ in the first step, the surface of Mn-doped Zn_2_GeO_4_ NPs was functionalized with amino groups to further attach lysozyme-binding aptamers, and finally these PersLNPs were introduced as lysozyme biosensors in the serum of cancer patients. The same type of PersLNPs functionalized with carboxyl groups and further treated with 1-ethyl-3-[3-(dimethylamino)­propyl]­carbodiimide and *N*-hydroxysuccinimide to form active esters on their surface enabled fingerprint imaging.^12^ The possibility of attaching the specific aptasensor at the PersLNPs surface for the detection of virus species was also presented.^13^ In another strategy, Mn-doped Zn_2_GeO_4_ NPs were functionalized with poly­(acrylic acid) and used in proof-of-concept sensing experiments on a persistent luminescence (PersL)-based sandwich structure.^14^ Most of the surface functionalization methods reported, however, refer to well-established protocols that are also used for other types of nanomaterials.^15^ Here, we decided to use a new strategy to enhance the biological activity and cellular uptake of Mn-doped Zn_2_GeO_4_ NPs, based on the deliberate introduction of a so-called artificial protein “corona” on their surface. In principle, when any kind of NPs are introduced to the biological environment, they are spontaneously surrounded by a layer of biomolecules, which in the next step determines their interaction with cells^16,17^ and can even spontaneously target the NPs to specific cell types.^18^ This activity is closely related to the presence of appropriate receptors on the surface of the target cells, e.g., cancer ones, which commonly interact with ligands present on the layered and functionalized NP surface. Based on these phenomena, an alternative approach has been then proposed for the surface functionalization of various types of NPs, where biologically important molecules are attached to the NPs surface prior to their introduction to the physiological environment, forming “artificial corona”.^19,20^ According to recent research concepts, organic-virus-derived structures with the ability to self-assemble on the inorganic NPs surface can be used to form such an “artificial corona”. As a result, the special proteins combined with NPs are able, as a whole, to mimic the shape, surface charge, and size of a virus particle. However, because they lack genetic material, these functionalized virus-like particles (VLPs) are unable to infect the host cell. On the other hand, expression and self-assembly of viral structural proteins can occur in various live and extracellular expression systems.^21,22^ The main role in the VLP interactions, recognition, and activation of the innate immune system is played by Toll-like receptors (TLRs)^23^ i.e., pillars to innate immunity and inflammation that are expressed not only in an innate immune system but also in various cancer cells because they have been linked to several forms of malignancy, including lung, breast, colon, and especially pancreatic tumors.^24^ In such an approach, the virus protein “artificial corona” formed at the NPs surface can be used as an efficient targeting factor.

Building on the above concept, in this paper, we functionalize the surface of rodlike, Mn^2+^-doped Zn_2_GeO_4_ NPs, recently often studied in the frame of different applications of SARS-CoV-2 spike protein.^25–27^ In that manner, the obtained PersLNPs with virus protein “artificial corona” remain noninfectious but could mimic the features of a real viral molecule, i.e., guide the functionalized NPs toward selected cells in which there is overexpression of TLRs. The size of synthesized Zn_2_GeO_4_:Mn^2+^ NPs was in the range between 10 and 300 nm, falling well with in the size scale of real virus species but adding the new optical functionality of strong persistent green luminescence. The functionality of the layered PersLNPs obtained via the selected SARS-CoV-2 S1 spike protein self-assembly was verified as biocompatible and effective in the interaction with model human pancreatic carcinoma BxPc cells, positive for TLRs (Scheme 1), while they were only minimally taken up by the cells lacking specified TLRs (i.e., Jurkat). Appropriate tailoring of the PersLNPs not only minimizes interference between NPs and surface functionalities but also promotes synergism among them and imparts new properties to the advanced final VLPs. Thus, these designed and engineered, bioinspired nanomaterials could be considered as novel VLPs: “smart”, multifunctional, and safe to use as optical agents.

**Scheme 1:**
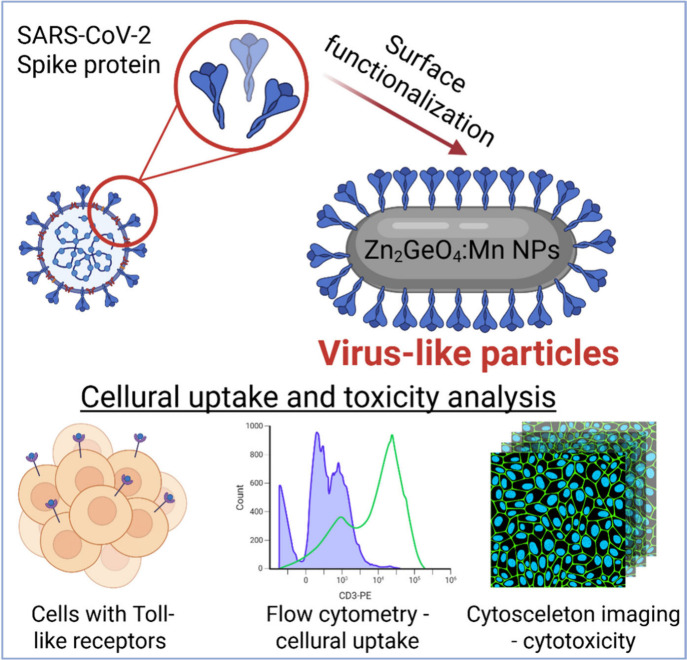
Schematic Representation of the Surface Functionalization of Synthesized Zn_2_GeO_4_:Mn^2+^ NPs, together with Visualization of Performed Biological Experiments

## 2 Experimental Section

### 2.1 Zn_2_GeO_4_:Mn^2+^ NPs Synthesis

A series of Zn_2_GeO_4_:Mn^2+^ NPs were obtained based on a previously published protocol.^11^ Needed chemicals, i.e., zinc nitrate hexahydrate [Zn­(NO_3_)_2_·6H_2_O (98%)], germanium­(IV) oxide [GeO_2_ (≥99.99%)], manganese nitrate [Mn­(NO_3_)_2_·*x*H_2_O (98%)], and sodium hydroxide [NaOH (≥98%)], were purchased from Merck and Sigma-Aldrich. Nitric acid [HNO_3_ (65% P.A.)] was supplied by Avantor Performance Materials Poland S.A., whereas ammonium hydroxide [NH_3_·H_2_O (25% P.A.)] was supplied by STANLAB.

First, 2 mmol of Zn­(NO_3_)_2_·6H_2_O, 0.005 mmol of Mn­(NO_3_)_2_·*x*H_2_O, and 300 μL of HNO_3_ were added to the glass vessel, containing 11 mL of water. In the meantime, a solution of Na_2_GeO_3_ was prepared by mixing 1 mmol of GeO_2_ with 2 mmol of NaOH dissolved in 1 mL of distilled water, followed by addition to the reaction mixture. Then, under intense mixing conditions, the pH of the reaction mixture was set to the desired value with the usage of NH_3_·H_2_O (i.e., 6.0, 7.0, 7.5, 8.5, and 9.5 for given synthesis, respectively) and left to stir at room temperature for 1 h. The exact pH of the reaction solution was monitored with a Mettler Toledo FiveEasy F20 pH meter. Finally, the reaction mixture was transferred to a Teflon vessel, placed in a microwave reactor (ERTEC Magnum VII, Poland), and the reaction was set to 220 °C for 4 h. Afterward, the obtained Zn_2_GeO_4_:Mn^2+^ NPs were then washed three times with deionized water by centrifugation (8.000 rpm) for 5 min, followed by dispersion in 10 mL of water.

### 2.2 Surface Modification of Selected Zn_2_GeO_4_:Mn^2+^ NPs with S1 SARS-CoV-2 Protein

Recombinant SARS-CoV Spike S1 Subunit His-tag Protein, CF, as well as Recombinant SARS-CoV-2 S GCN4-IZ Alexa Fluor 488 Protein, were purchased from BioTechne (Minneapolis, MN). For surface modification, we chose Zn_2_GeO_4_:Mn^2+^ NPs showing the most intense visible emission under UV excitation (i.e., sample obtained at pH 9.5), and the procedure was carried out based on the protocol presented in a previously published paper.^28^ A solution of Zn_2_GeO_4_:Mn^2+^ NPs with a concentration of ca. 8 × 10^9^ particles/mL in water was prepared.

In order to ensure the required ratio between the amount of PersLNPs and the concentration of S1 SARS-CoV-2 protein in the solution, first, the average mass value per mL (g/mL) of PersLNPs was estimated by the evaporation and weighing of a certain amount of stock solution, using the RADWAG MYA 2.4Y scale. Then, based on transmission electron microscopy (TEM) images, the mean length and width of PersLNPs were calculated, and by assuming a cuboidal shape, the volume of a single NP was approximated. With the density of Zn_2_GeO_4_ (4.56 g/cm^3^) known, next the mass of a single NP was calculated. Finally, when the previously determined mass of PersLNPs in 1 mL was divided by the mass of a single PersLNP, the approximate value for the g/mL concentration of PersLNPs in 1 mL was determined.

To 500 μL of the selected Zn_2_GeO_4_:Mn^2+^ NPs solution with the above-mentioned concentration was added 500 μL of S1 SARS-CoV-2 protein solution, dissolved in PBS, of prepared specific C1–C4 concentrations (i.e., 5, 10, 15, and 20 μg/mL, respectively), resulting in 1 mL total. Such combined mixtures were then stirred for 1 h at room temperature, and finally Zn_2_GeO_4_:Mn^2+^ NPs with the S1 SARS-CoV-2 spike protein attached to the surface were obtained. The same procedure was used to functionalize the surface of Zn_2_GeO_4_:Mn^2+^ NPs with Alexa Fluor 488 stained S1 SARS-CoV-2 spike protein for flow cytometric analysis. Finally, the amount of S1 SARS-CoV-2 spike protein attached per a single Zn_2_GeO_4_:Mn^2+^ NP was estimated based on absorption measurements (see the Supporting Information for the details).

### 2.3 Modification of SARS-CoV-2 S1 Surface-Functionalized Zn_2_GeO_4_:Mn^2+^ NPs with SARS-CoV-2 Antibodies

In order to attach antibodies to the S1 SARS-CoV-2 protein present at the Zn_2_GeO_4_:Mn^2+^ NPs surface and thus confirm their efficient attachment to the PersLNPs surface, a 500 μL solution of surface-modified Zn_2_GeO_4_:Mn^2+^ NPs (with selected C4 protein concentration) was mixed with a prepared solution of 500 μL of S1 SARS-CoV-2 antibodies of the exact same concentration [20 μg/mL concentration (w/w = 1:1)] and left to stir for another 1 h in room temperature.

### 2.4 ζ-Potential Measurements

All ζ-potential measurements were carried out via the electrophoretic light scattering (ELS) method, within ambient air at 25 °C, with a Malvern Instruments Zetasizer Nano Pro. Each of the results obtained was based on three, following averaged measurements.

### 2.5 Protein Analysis: Electrophoresis

The protein and Zn_2_GeO_4_:Mn^2+^ NPs complex was analyzed using nondenaturating polyacrylamide gel electrophoresis (PAGE) analysis. PAGE was performed by using final acrylamide concentrations of 12% and 5% (w/v) for separating and stacking gels, respectively. After electrophoresis, the protein bands were stained with BlueStain Sensitive stain (EurX Cat. No. E0298-01).

### 2.6 Crystal Structure and Morphology Characterization

The crystal structure of as-synthesized Zn_2_GeO_4_:Mn^2+^ NPs was defined by a powder X-ray diffraction (PXRD) approach via Bragg–Brentano geometry PROTO AXRD with Cu Kα_1_ (30 kV, 20 mA) radiation within the 10–70° 2θ range. TEM images were taken to characterize the morphology by either a FEI Tecnai G^2^ 20 XTWIN (200 keV) microscope or a ThermoFisher Scientific Talos F200i (200 keV) microscope.

### 2.7 Spectroscopic Properties Evaluation

All measurements of the optical properties were conducted at room temperature and in ambient air, except for the thermoluminescence (TL) curves, which were recorded from 25 to 300 °C within the spectral range 460–610 nm using a HC 535/150 band-pass filter. The Stokes emission upon UV excitation, λ_excitation_ = 255 nm, and excitation spectra for wavelength-observed λ_emission_ (λ_em_) = 530 nm were measured with a FluoroMax-4 Horiba spectrofluorometer for colloidal dispersions. PersL spectra and TL curves were recorded for samples in the form of dried powders, with Lexsyg research with a fully automated TL/OSL reader from Freiberg Instruments GmbH. As an irradiation source, a Kamush LP254/366UV 6 W lamp (254 nm) was used. The TL glow curves were recorded with an R13456 photomultiplier tube (Hamamatsu Photonics). PersL spectra were acquired with an Andor DU420A EM-CCD camera (Oxford Instruments). Before measurements, all samples were preheated to 350 °C and held for 60 s to release carriers already trapped and prepare samples for their selective excitation using UV light. Each sample was then irradiated with a UV lamp, and PersL spectra were collected after a 1 min pause following the end of irradiation. TL curves were then recorded 10 min after irradiation at a heating rate of 1 °C/s.

### 2.8 Biological Activity

The BxPC-3 cells (human pancreatic cancer cell lines, CRL-1687), purchased from ATCC, were derived from a 61-year-old female in 1986. The cells were maintained in culture flasks with a surface area equal to 75 cm^2^ (Falcon Cell Culture Flasks) in Dulbecco’s modified Eagle medium (DMEM, IITD, Wroclaw, Poland) supplemented with 10% fetal bovine serum (FBS, Gibco) and 50 μg/mL penicillin and streptomycin (Sigma-Aldrich, Poznan, Poland). Jurkat cells—acute T cell leukemia (Clone E6-1, TIB-152), an immortalized T-lymphocyte cell line—were purchased from ATCC. The cultures were incubated in a humidified atmosphere with 5% CO_2_ at 37 °C. The cells were grown in a suspension in a RPMI-1640 medium (IITD, Wroclaw, Poland). Jurkat cells were passaged by centrifugation and removal of the digested medium.

Flow cytometric analysis was performed to assess the ability of the studied Alexa Fluor 488-stained S1 SARS-CoV-2 spike protein-functionalized Zn_2_GeO_4_:Mn^2+^ NPs to be uptaken by the BxPC-3 (positive for TLRs) and Jurkat (negative for TLRs) cells. The cells (density of 4 × 10^5^) were seeded on 24-well plates and left to adhere overnight. Each Zn_2_GeO_4_:Mn^2+^ NP was added in a ratio of 1:50 and diluted in a culture medium. Then, the cells were incubated for 2 h at 37 °C. After washing in PBS (not containing calcium and magnesium ions, IITD, Poland), they were trypsinized and resuspended in 0.5 mL of PBS (BioShop, EPRO, Poland). The flow cytometric measurements were performed on a CyFlow Cube 6 flow cytometer (Sysmex, Poland). The fluorescence of the extract was measured with a FL-3-H detector. A total of 10000 events were measured in triplicate from each sample. Data were collected and analyzed with *CyView* software (Sysmex, Poland).

An Olympus BX53 fluorescence microscope was used to visualize the actin filaments and nuclei of the cells studied. For this experiment, the BxPC-3 cells were seeded directly on 18-mm-diameter round microscope coverslips (Thermo Fisher Scientific Inc.) in 6-well plates (Sarstedt, EquiMed, Poland) and adhered for 24 h. At a later stage, the cells were treated either with the S1 SARS-CoV-2 spike protein functionalized or with the uncoated Zn_2_GeO_4_:Mn^2+^ NPs dispersions. Following 24 h of incubation, the cells were washed twice with phosphate-buffered saline (PBS, BioShop, EPRO, Poland), fixed in 4% paraformaldehyde (Polysciences, Inc., Bergstrasse, Germany) for 10 min, and washed again with PBS. The actin cytoskeleton was stained with Alexa Fluor546 Phalloidin following the manufacturer’s protocol (Thermo Fisher Scientific Inc.). Fluorshield with 4,6-diamidino-2-phenylindole (DAPI, fluorescent DNA-binding dye) was applied to visualize the nuclei and mount the cells after excitation at 405 nm.

## 3 Results and Discussion

### 3.1 Morphology Characteristics

Parts a–f of Figure 1 provide an overview of the NPs morphology based on TEM images of Zn_2_GeO_4_:Mn^2+^ NPs synthesized by setting different pH values of the reaction mixture. The imaging results confirm the nanoscale size as well as rodlike structure preserved for Zn_2_GeO_4_:Mn^2+^ NPs obtained at each of the pH values set during the syntheses, except for the size of the material obtained at pH 6.0. Additionally, TEM imaging allowed us to perform size measurements, followed by analysis of the size distribution histograms (Figures S2 and S3 and Table S1). The largest size regarding the length and width was recorded for the lowest pH used during the series of syntheses: pH 6.0, i.e., 1527 and 334 nm, respectively. Within the pH increase, a significant decrease in the size can be noted for both the length and width values. The measured size of PersLNPs obtained at pH 7.0 was equal to 130 nm for length and 23 nm for width. For the range from pH 7.5 to 9.5, according to the length, it could be observed that PersLNPs are characterized by length sizes from 70 nm (pH 7.5) to 88 nm (pH 8.0). For the highest pH 9.5 set during the synthesis process of Zn_2_GeO_4_:Mn^2+^ NPs, the measured length is around 76 nm. The smallest particles have been obtained for pH 8.0, resulting in 67 nm length. A similar tendency is observed for width measurements. For Zn_2_GeO_4_:Mn^2+^ NPs synthesized in the range from pH 7.0 to 9.5, measured values are in the range of 23 nm for PersLNPs synthesized at pH 7.0 to 17 nm for those at pH 9.5. The width of the materials obtained at pH 7.5, 8.0, and 8.5 were defined as 17, 21, and around 20 nm, respectively. The measured PXRD spectra presented in Figure 1g prove the presence of the desired and pure-phase crystal structure of rhombohedral [Inorganic Crystal Structure Database (ICSD) file No. 16173][Bibr ref29] Zn_2_GeO_4_ for all of Zn_2_GeO_4_:Mn^2+^ NPs obtained. Structural and morphology studies therefore present significant similarities compared to the mentioned work,^11^ based on which series of current syntheses were carried out. For both works, a rodlike structure of the obtained Zn_2_GeO_4_:Mn^2+^ NP is present, and a major drop in size occurs for PersLNPs obtained at pH 7.0 and above. Additionally, crystal structure analysis via PXRD confirms the presence of the rhombohedral crystal phase.

**Figure 1:**
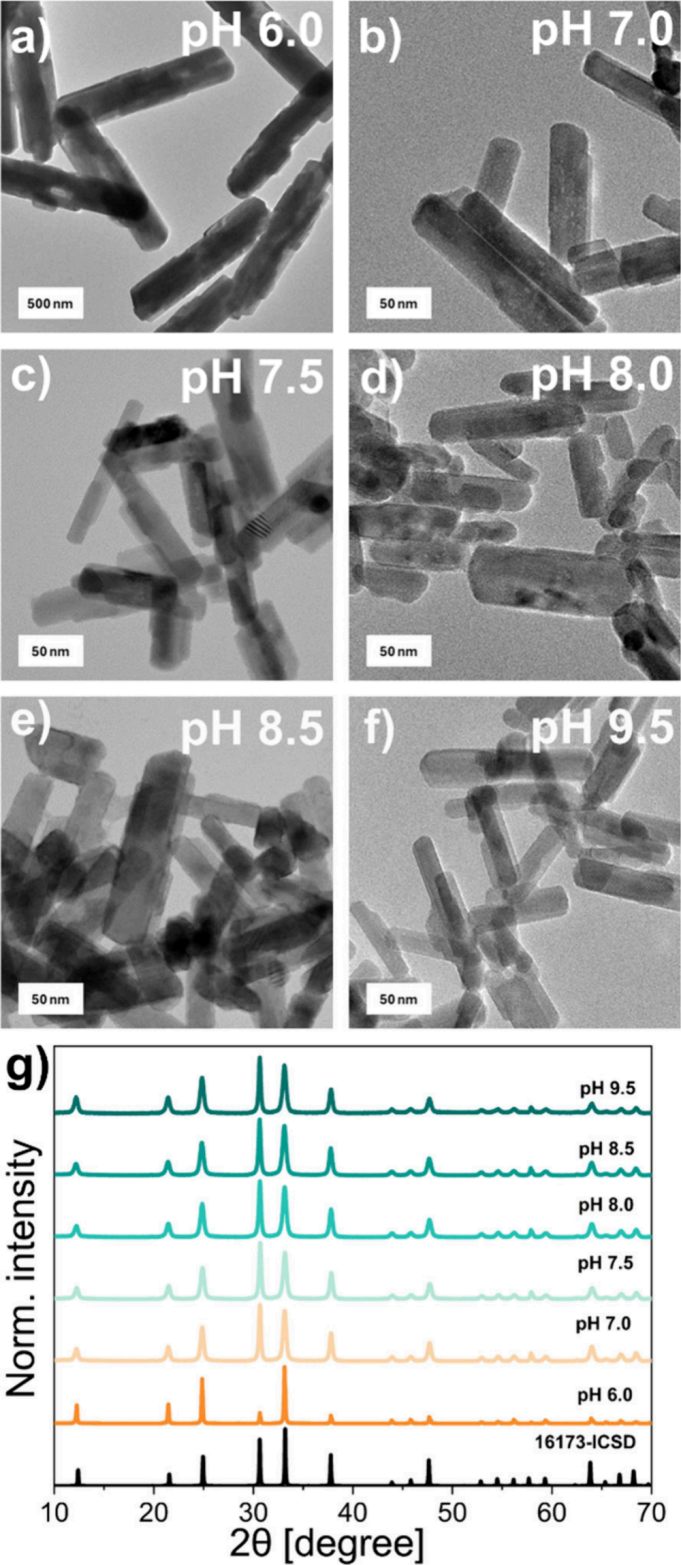
(a–f) TEM images of Zn_2_GeO_4_:Mn^2+^ NPs obtained for various pH values during the synthesis, i.e., 6.0, 7.0, 7.5, 8.0, 8.5, and 9.5, respectively. (g) PXRD patterns of Zn_2_GeO_4_:Mn^2+^ NPs obtained for different pH values set during the synthesis, compared with the standard 16173-ICSD pattern.

### 3.2 Spectroscopy Analysis

Crystal structure and morphology studies were followed by optical properties characteristics. Figure 2a presents emission spectra of the obtained Zn_2_GeO_4_:Mn^2+^ NPs under 255 nm UV excitation. It can be observed that the higher the pH set during the synthesis, the more intense the emission that occurs, peaking at λ = 530 nm, which for this wavelength can be assigned to Mn^2+^ ion transition of ^4^T_1_ → ^6^A_1_,^30–32^ i.e., energy transfer through the matrix followed by deexcitation to the Mn^2+^ ground state.^14^ On the other hand, the lower the pH set, the less intense the emission that is noted in the mentioned wavelength region and the more the emission of intrinsic defects that starts to build up in the spectral range from λ = 400 nm up to λ = 530 nm, occurring as visible, bluish, broad emission bands (probe pH 7.0–8.5).^31^

**Figure 2:**
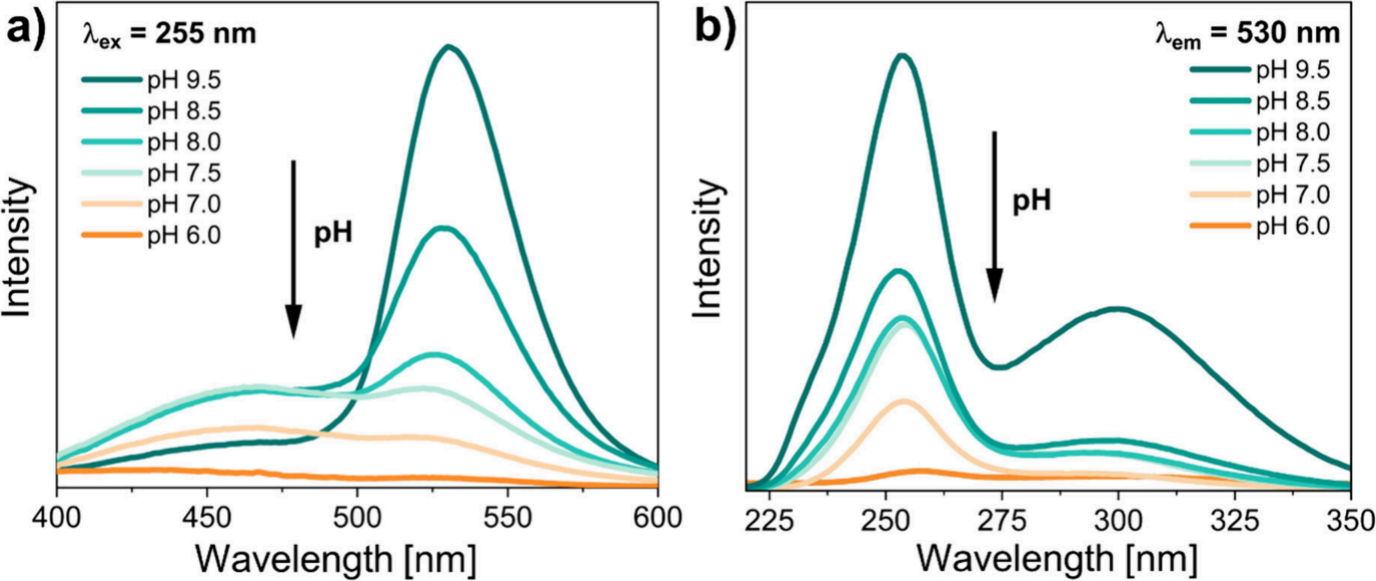
(a) Emission spectra of Zn_2_GeO_4_:Mn^2+^ NPs obtained at different pH values set during synthesis under λ = 255 nm excitation and (b) excitation spectra of the exact same series of materials for λ = 530 nm.

Figure 2b points out the combined excitation spectra for the λ_em_ = 530 nm wavelength being observed. As for the emission spectra, pH dependency is clearly visible. The higher the pH of the synthesis reaction set, the more intense the excitation band observed for λ_ex_ = 255 nm. Based on the reported literature, the excitation intensity in the 254 nm region can be assigned to the energy transition occurring from the valence band to the conduction band within the Zn_2_GeO_4_ matrix itself and afterward to the Mn^2+^ ions, whereas excitation band peaking at 300 nm can be attributed to the charge transfer to Mn^2+^ ions.^14,33^ Emission and excitation spectra characteristics were investigated first, and the PersL decay time measurements were studied thereafter, performed at room temperature; successively, TL measurements were taken. Parts b–g of Figure 3 show the results obtained for the whole series of Zn_2_GeO_4_:Mn^2+^ NPs with calculated average PersL decay time values for each material. A clear tendency can be identified in that the longest PersL decay times in room temperature (τ_1/2_) are measured for PersLNPs obtained in lower pH values, i.e., pH 6.0–7.5 (that is, 87.52, 86.26, and 75.61 s, respectively), whereas for materials synthesized in pH 8.0–9.5, the measured PersL decay times are gradually shorter, yet still hovering around 70 s (for pH 8.0, 69.29 s, for pH 8.5, 67.76 s, and for pH 9.5, 70.60 s). The Han group measured a similar τ_1/2_ = 70.9 s decay value for Zn_2_GeO_4_:Mn^2+^ NPs nanocrystals,^34^ preceded by UV excitation. Yet, it needs to be underlined that the PersL properties of any material strongly depend on the morphology, i.e., structural defects that are responsible for energy storage and further the release via charge trapping.^35^ The morphology properties are a direct result of the synthesis, where parameters such as the temperature or time can affect the size and shape of the resulting material. Taking into account those factors, it is challenging to directly compare the optical properties between different synthesis approaches. Based on analysis of the PersL decay curves (Figure 3b–g), it can be annotated that, even though different τ_1/2_ values for each pH value used during the synthesis for Zn_2_GeO_4_:Mn^2+^ NPs have been measured (Table S1), the decay curves for the whole batch seem to be significantly quenched around 200 s after measurement has started. By comparison with the Tan group work,^11^ due to the exact same synthesis protocol, notably the decay curves are different; in their measurements, it can be distinguished that, for lower pH values (i.e., 6.0 and 7.0), PersL seems to be completely quenched by the 100 s mark and ca. 150 s, respectively. For higher pH values, this time shifts toward higher values; for pH 7.5, it is around 300 s, for pH 8.0, it is 400 s, and for pH 8.5 and 9.5, it extends beyond 400 s. However, no exact values of τ_1/2_ were given in the mentioned work. Further, to evaluate the PersL decay, TL measurements were performed, as outlined in Figure 3a. All of the materials exhibit only one noticeable peak. The lower the pH set during the synthesis, the more intense the emission that is observable for all samples in the 60–80 °C temperature region. In general, TL measurement is a tool that can be used to evaluate the trap characteristics within a given matrix, i.e., the amount of the charge carriers as well as depth distribution; thus, measurement results reveal data regarding the defect traps and allow one to analyze the PersL properties.^36^ The longest PersL for Zn_2_GeO_4_:Mn^2+^ NPs synthesized at pH 6.0 corresponds in an expected manner to the most intense emission measured for the TL curve. Yet, at the same time, for higher pH set during the synthesis, there is visible a slight shift in the temperature at which the intensity peak is present toward lower temperature compared to the most intense one measured for pH 6.0, which occurs for 70 °C (343 K) and is recognizable in Figure 3a. For Zn_2_GeO_4_:Mn^2+^ NPs, it is possible to find in the literature similar reports, where the peak for TL measurements is present around 337 K.^37^ Based on TL measurements, trap depth (TD) values can be very roughly estimated via Urbach’s equation:^38^

1 $$\mathrm{TD}=\frac{T}{500}\;\mathrm{eV}$$

where *T* (K) stands for the temperature at which the peak of the emission intensity is present. The differences in the calculated TD values (based on eq 1) for Zn_2_GeO_4_:Mn^2+^ NPs obtained at different pH levels during the synthesis process are not significant and fall into the 0.67 up to 0.69 eV range (Table S1), which can be considered to be the so-called shallow traps.^32^ Thus, the energy from these shallow traps is most probably the main component of the observed long PersL decays in ambient conditions, for the whole series of Zn_2_GeO_4_:Mn^2+^ NPs (Figure 3b–g) obtained. Similar conclusions regarding shallow traps and their contribution to room temperature PersL emission can be found in ref 39, where authors established that the traps occurring around 70 °C (343 K) were the main contributors to PersL observed at room temperature.

**Figure 3:**
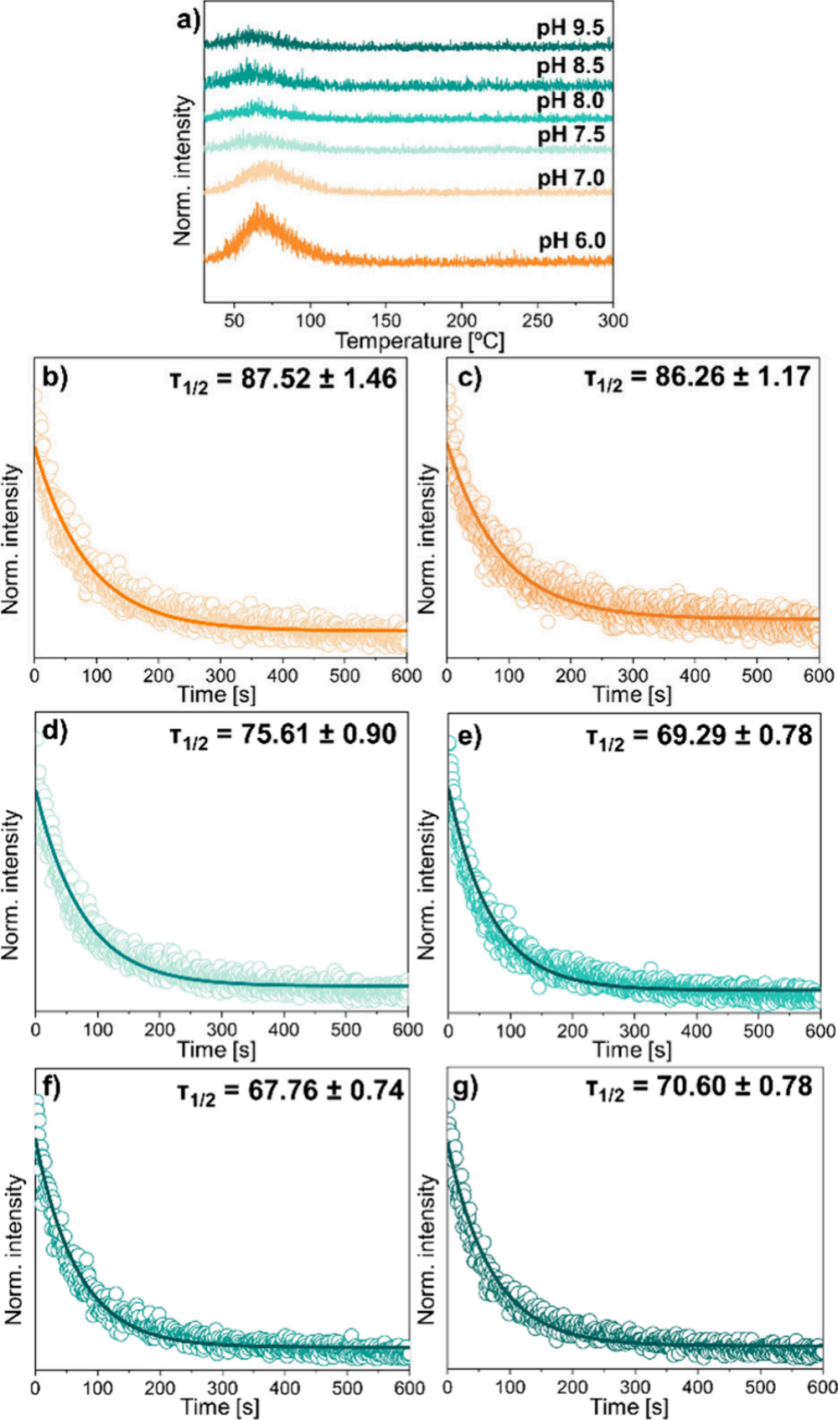
Measured TL curves (a) combined with PersL decay curves (b–g) measured for Zn_2_GeO_4_:Mn^2+^ NPs obtained in different pH values (i.e., 6.0, 7.0, 7.5, 8.5, and 9.5, respectively) after UV irradiation for 5 min.

### 3.3 SARS-CoV-2 Surface Modification of Zn_2_GeO_4_:Mn^2+^ NPs Characteristics

After measurements of the morphology, crystal structure, and optical properties of the synthesized batch of Zn_2_GeO_4_:Mn^2+^ NPs, the most promising candidate was selected for further functionalization toward biological applications with SARS-CoV-2 S1 spike protein. The measured size of Zn_2_GeO_4_:Mn^2+^ NPs synthesized at pH 9.5 was around 75 nm (Figures 1f and S4a,b), whereas the size of SARS-CoV-2 virus particles resolve around 100 nm.^28,40^ Thus, given their similar sizes, coating the surface of Zn_2_GeO_4_:Mn^2+^ NPs with S1 spike protein of SARS-CoV-2 (without harmful mRNA) allowed one to create advanced functional materials, which in the literature are considered to be VLPs.^28^ These Zn_2_GeO_4_:Mn^2+^ NPs, having unique optical properties, including strong green luminescence and PersL resolution at a decay time of τ_1/2_ = 75 s, coated with a layer of proteins of the virus, can therefore in a safe way imitate real virus molecules. Such a feature may allow the study of the relationship of interactions of a living organism with surface-functionalized Zn_2_GeO_4_:Mn^2+^ NPs, for example, via bioimaging. The goal of our research was also to see whether such surface modification with the S1 spike protein of SARS-CoV-2 would have a beneficial effect on reducing the cytotoxicity of the Zn_2_GeO_4_:Mn^2+^ NPs themselves to the environment of the cell in which they will be found and also whether it would confer specificity to such modified materials, for example, for targeted therapy applications.^21^ The colloidal stability of PersLNPs before and after functionalization was studied with an ELS technique (Figure S5), which indicated that unmodified Zn_2_GeO_4_:Mn^2+^ NPs present the highest value of the ζ potential: −34.43 ± 0.34 mV. Such a high electrical potential assures the colloidal stability of particles due to electrostatic repulsion. In the next step, determination of the ζ potential from the electrophoretic mobility of the as-synthesized and further functionalized NPs dispersed in the dispersing media was used as an indicator of successful surface modification. Because the charge of the SARS-CoV-2 S1 spike protein in a PBS solution was positive, we assumed simple physical interaction between inorganic NPs and proteins based on electrostatic force. Within the increase of the SARS-CoV-2 S1 protein concentration used for surface modification, the increase in the ζ-potential value can be noted, i.e., for 5 μg/mL, the measured value is equal to −27.86 ± 0.49 mV, for 10 μg/mL, it is −27.92 ± 0.94 mV, and for 15 μg/mL, it corresponds with −24.20 ± 0.39 mV, whereas the 20 μg/mL measured value is −14.07 ± 0.65 mV. Thus, changes in the value of the ζ potential represent that the electrophoretic mobility of Zn_2_GeO_4_:Mn^2+^ NPs has changed, following increasing concentration of the SARS-CoV-2 S1 protein used and thus indirectly proving the attachment of those molecules to the surface of NPs. Additionally, the evolution of the ζ potential during surface functionalization was found to be in line with TEM imaging, which showed an evident organic coating on the NPs’ surface after functionalization. With reference to Andrzejewska’s work,^28^ where authors have formed S1 protein corona on Au NPs coated with cetyltrimethylammonium bromide, they noted a decrease in the ζ potential from −44.42 ± 3.67 to −10.00 ± 1.75 mV. The outcome of our surface modification, with a registered decline in the electrokinetic potential from −34.34 ± 0.34 mV for the reference sample to −14.09 ± 0.65 mV for the sample functionalized with the highest protein concentration (20 μg/mL), illustrates thus the approximate ζ-potential change of the Zn_2_GeO_4_:Mn^2+^ NPs with respect to the protein used. Additionally, the Zn_2_GeO_4_:Mn^2+^ NPs–SARS-CoV-2 protein complex was studied with gel electrophoresis, additionally confirming the presence of desired molecules at the NPs surface (Figure S6).

In the next step, the appearance of “artificial protein corona” at the Zn_2_GeO_4_:Mn^2+^ NPs surface upon functionalization was visualized based on TEM imaging, which showed visible organic coating around Zn_2_GeO_4_:Mn^2+^ NPs achieved for all SARS-CoV-2 S1 spike protein concentrations used during the surface modification process, i.e., 5, 10, 15, and 20 μg/mL (Figure 4b–e), in comparison to an unmodified reference sample (Figure 4a). The modified sample with the highest concentration (Figure 4d) of the proteins at the surface was then treated with S1 SARS-CoV-2 antibodies, in order to additionally confirm the efficient attachment of the proteins. For all of the SARS-CoV-2 spike protein concentrations used and the added antibodies, there is a visible thin organic coating around the inorganic Zn_2_GeO_4_:Mn^2+^ NPs. The coating can be distinguished from the Zn_2_GeO_4_:Mn^2+^ NPs based on the irregular, brighter shape compared to PersLNPs themselves. The measured thickness of this “artificial protein corona”, based on TEM images, for each concentration can be described as rather uneven between particles. On average, for 5 μg/mL, it is in the range from 3 to 6 nm, for 10 μg/mL from 2.5 to 4 nm, for 15 μg/mL from 3.5 to 14.3 nm, and for 20 μg/mL from 2.5 to 5 nm. The highest measured values of the organic coating are present for PersLNPs modified with both 20 μg/mL proteins and 20 μg/mL antibodies, where the thickness of the visible organic coating is in the range from 6.5 to 13 nm. It should be noted that the lower end of the mentioned range (ca. 2.5 nm) represents the tendency across the modified PersLNPs because this coating thickness is the most common on the surface of PersLNPs. Thus, it can be observed that, for all concentrations used, the values remain similar, around 2–3 nm. The higher end of the range can change significantly, depending on how accumulation of the organic part of the proteins on the surface has occurred, whereas this phenomenon can be observed in TEM images (Figures 4b–f and S4c–j). For Zn_2_GeO_4_:Mn^2+^ NPs first functionalized with SARS-CoV-2 S1 spike proteins (concentration C4) and then additionally modified with antibodies, we observe an increase in the range of coating, especially the lower end, from 2–3 nm up to 6.5 nm. Therefore, this suggests that the antibodies have successfully attached to the SARS-CoV-2 S1 spike proteins that formerly attached at the surface of Zn_2_GeO_4_:Mn^2+^ NPs, resulting in a measured increase in the thickness size.

**Figure 4:**
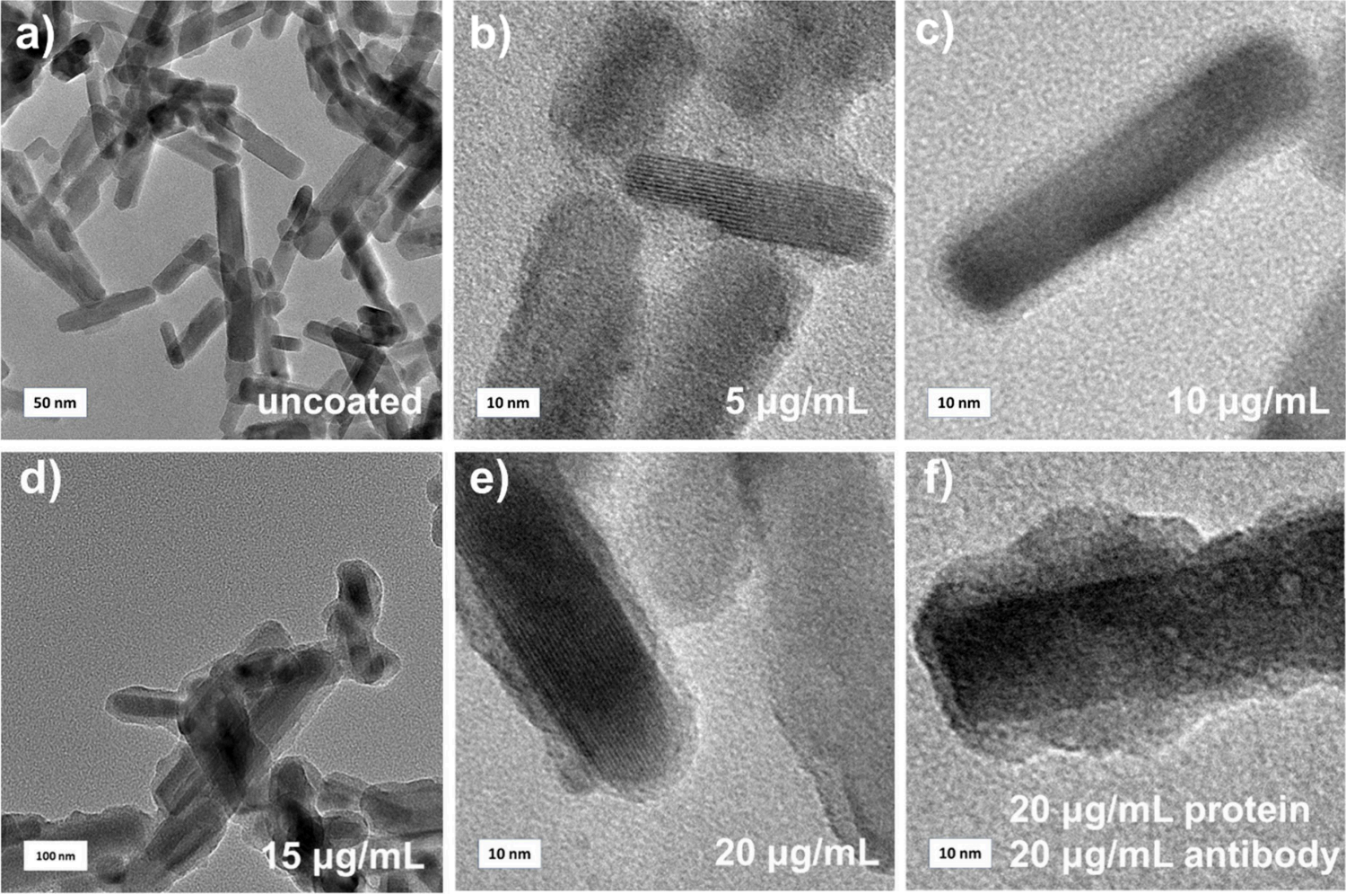
TEM images of Zn_2_GeO_4_:Mn^2+^ NPs obtained in pH 9.5: (a) uncoated; (b–e) Zn_2_GeO_4_:Mn^2+^ NPs modified with different S1 SARS-CoV-2 protein concentrations (i.e., 5, 10, 15, and 20 μg/mL, respectively); (f) Zn_2_GeO_4_:Mn^2+^ NPs modified with 20 μg/mL of S1 SARS-CoV-2 S1 protein after the addition of SARS-CoV-2 antibodies of 20 μg/mL concentration.

### 3.4 Optical Properties of Surface-Modified Zn_2_GeO_4_:Mn^2+^ NPs

The surface modification and morphology studies via TEM imaging were followed by reexamination of the optical properties of functionalized PersLNPs in order to ensure that the desired spectroscopic features are kept after surface functionalization. Figure 5a presents the luminescence spectra of surface-functionalized materials compared to unmodified Zn_2_GeO_4_:Mn^2+^ NPs, therefore confirming that surface modification did not quench the ability of these studied NPs to exhibit green emission under UV excitation and herein preserving the unique optical properties, including long afterglow. Parts b–e of Figure 5 display the properties of PersL of surface-modified Zn_2_GeO_4_:Mn^2+^ NPs. The prolonged decay curves are clearly visible, yet the measured average PersL decay times are slightly quenched by the presence of proteins at the surface, i.e., concerning τ_1/2_ of unmodified material = 70.60 s; for 5 μg/mL of the SARS-CoV-2 spike protein used for surface functionalization, τ_1/2_ = 63.53 s, quenched by 10.01%, for 10 μg/mL and τ_1/2_ = 48.77 s, it is quenched by 30.92%, for 15 μg/mL and corresponding τ_1/2_ = 55.13 s, it is equal to 21.91% quenching, and for 20 μg/mL and τ_1/2_ = 61.80 s, it is equal to 12.46% quenching.

**Figure 5:**
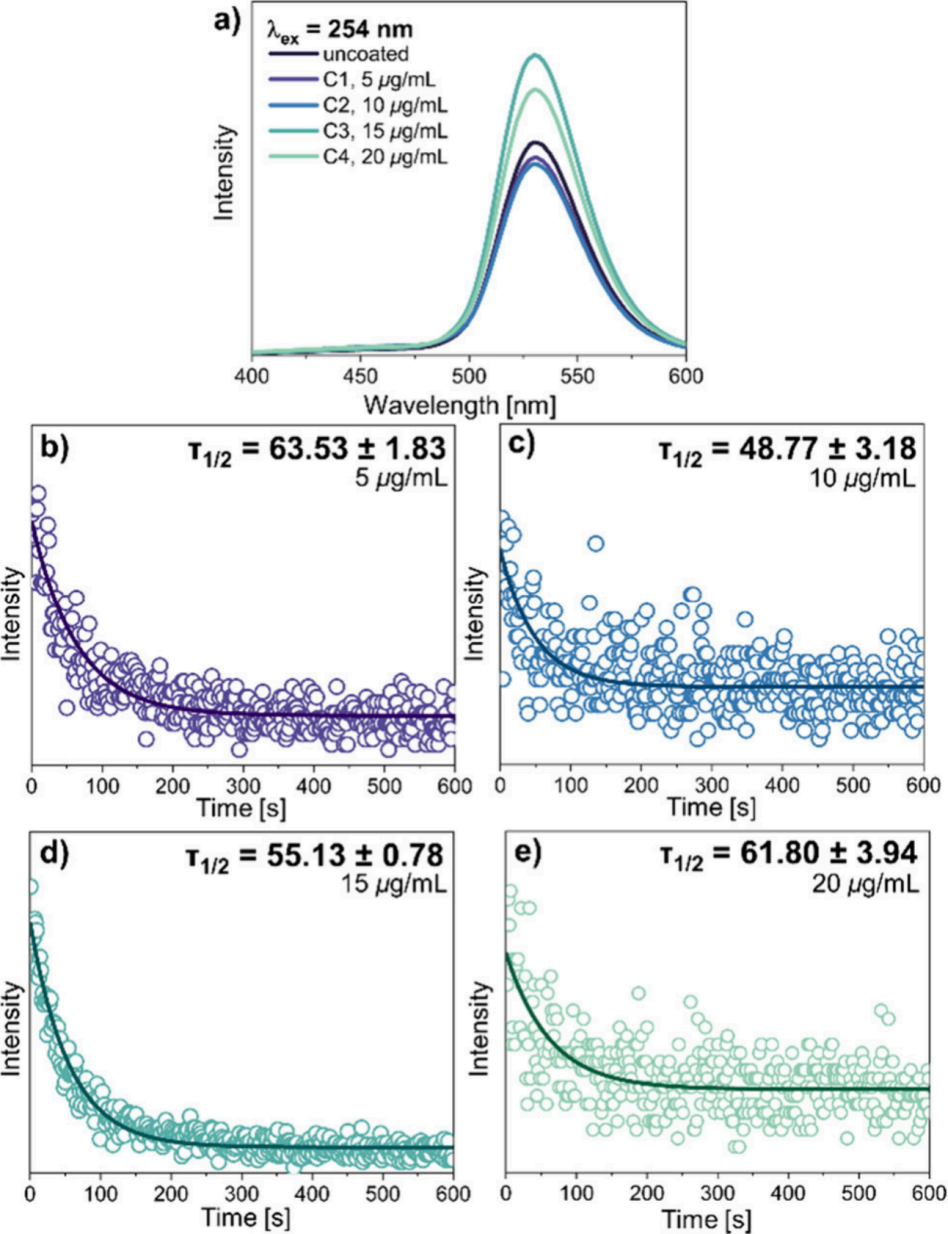
(a) Luminescence spectra under 254 nm excitation of Zn_2_GeO_4_:Mn^2+^ NPs modified with a S1 SARS-CoV-2 protein, obtained for different protein concentrations C1–C4, compared to uncoated Zn_2_GeO_4_:Mn^2+^ NPs obtained at pH 9.5 as a reference sample. (b–e) PersL decay curves measured for surface-coated Zn_2_GeO_4_:Mn^2+^ NPs for all S1 SARS-CoV-2 protein concentrations used.

### 3.5 Biological Activity

In the initial phase of the performed biological research, the cellular uptake of both functionalized and nonfunctionalized Zn_2_GeO_4_:Mn^2+^ NPs using flow cytometry was investigated (Figure 6). For that purpose, two control experiments were done; i.e., the cellular uptakes of uncoated Zn_2_GeO_4_:Mn^2+^ NPs and of PersLNPs with the attached S1 SARS-CoV-2 protein (concentration C4) were studied. In the designed control experiments, no additional dye was used, which should be detected by the flow cytometry measurement system. Finally, the cellular uptake of Zn_2_GeO_4_:Mn^2+^ NPs functionalized with an Alexa Fluor 488-stained SARS-CoV-2 S1 spike protein was studied to show the difference in the designed PersLNPs uptake in the cells with and without specific TLRs. Figure 6a illustrates the percentage of positively stained cells for the uptake of Zn_2_GeO_4_:Mn^2+^ NPs fuctionalized with SARS-CoV-2 S1 spike protein labeled with Alexa Fluor 488 in comparison to the above control groups, including uncoated Zn_2_GeO_4_:Mn^2+^ NPs and the sample functionalized with SARS-CoV-2 S1 spike protein but nonstained, using two distinct cancer cell lines: human T-cell leukemia Jurkat cells (Figure 6b) and pancreatic carcinoma BxPC-3 cells (Figure 6c). A key aspect was to assess cells with various TLRs expression in terms of uncoated and coated Zn_2_GeO_4_:Mn^2+^ NPs recognition and uptake. TLRs are essential components of the innate immune system, recognizing conserved molecular patterns from viral, bacterial, and fungal pathogens to trigger immune responses. Their activation induces the secretion of various pro-inflammatory cytokines, such as interleukin-1 (IL-1), IL-6, tumor necrosis factor-α (TNF-α), and type I interferons, which contribute to immune surveillance and inflammation-mediated tumor progression or suppression.^23,41^ Notably, TLRs are frequently overexpressed in multiple cancer types, including lung, breast, and pancreatic cancer, where their dysregulated activity influences tumor growth and immune evasion mechanisms.^42^ Performed measurements reveal insignificant uptake of uncoated and functionalized but nonstained Zn_2_GeO_4_:Mn^2+^ NPs, with no evidence of a difference between cell lines. A change is, however, noticed when Zn_2_GeO_4_:Mn^2+^ NPs functionalized with SARS-CoV-2 S1 spike protein labeled with Alexa Fluor 488 are used for the flow cytometry measurements, with an evident difference in functionalized Zn_2_GeO_4_:Mn^2+^ NPs uptake between the two studied cell lines present (Figure 6a). Jurkat cells, which lack TLRs overexpression, exhibited minimal uptake of the functionalized Zn_2_GeO_4_:Mn^2+^ NPs, as evidenced by the low percentage of positively stained cells similar to that of the control samples. In contrast, pancreatic BxPC-3 cells, known for their upregulated TLRs expression, displayed six times greater uptake of the functionalized Zn_2_GeO_4_:Mn^2+^ NPs compared to Jurkat cells. This suggests that the TLR-mediated endocytosis pathway significantly contributes to the internalization of SARS-CoV-2 S1 spike protein-functionalized Zn_2_GeO_4_:Mn^2+^ NPs in TLR-positive pancreatic cancer cells. These findings validate the successful functionalization of the PersLNPs surface and their potential application as virus-mimicking VLPs and additionally tumor-targeting agents. The preferential uptake by pancreatic cancer cells highlights their potential for further selective drug delivery, vaccine development, or theranostic applications, offering a promising avenue for targeted cancer therapies. Future studies should explore the mechanistic aspects of TLR-PersLNPs interactions, the downstream signaling pathways involved, and the implications of this uptake for enhancing anticancer immune responses.

**Figure 6:**
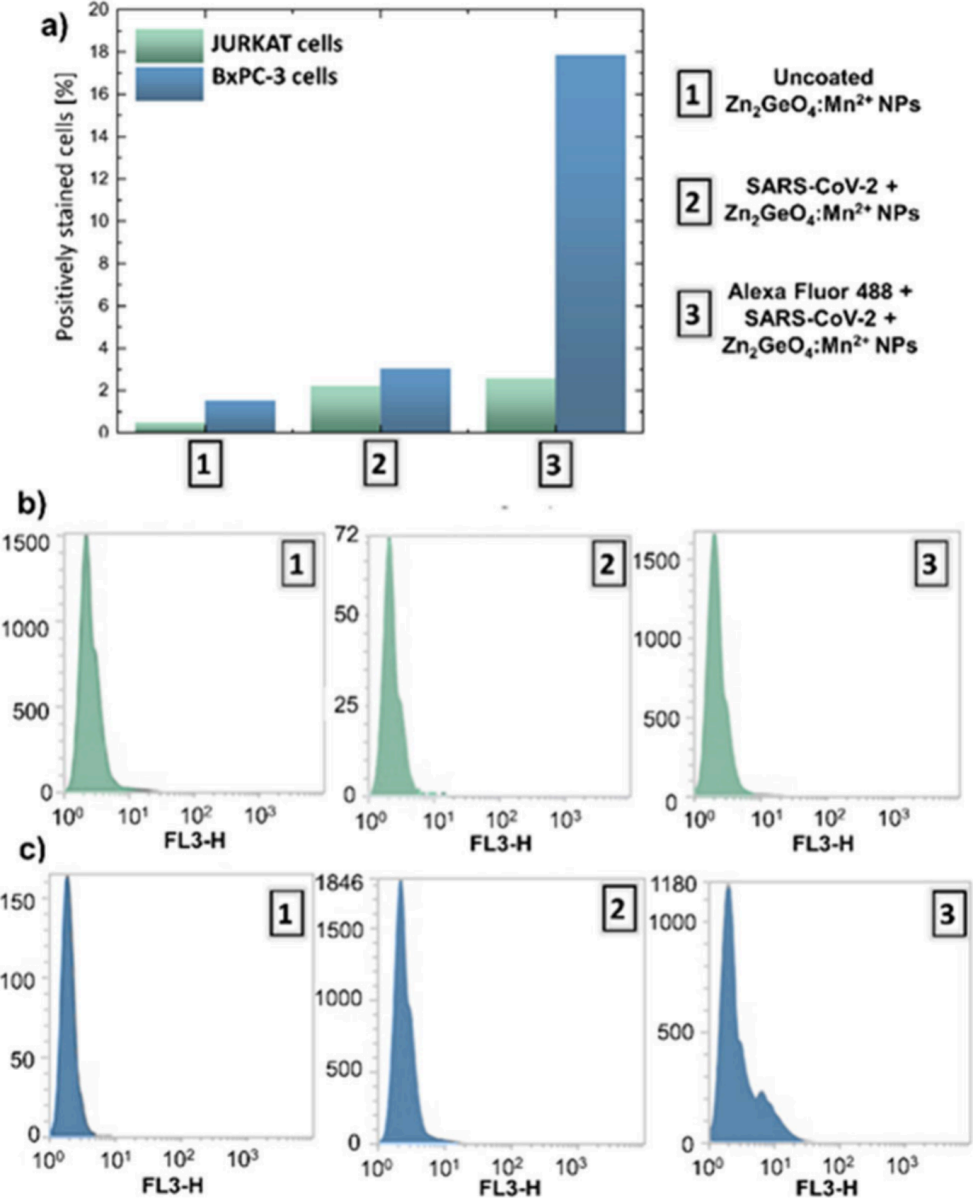
Flow cytometry uptake comparison for Jurkat and BxPC-3 cell lines after 2 h of incubation at 37 °C with uncoated Zn_2_GeO_4_:Mn^2+^ NPs (control), Zn_2_GeO_4_:Mn^2+^ NPs functionalized with SARS-CoV-2 S1 spike protein (control), and Zn_2_GeO_4_:Mn^2+^ NPs functionalized with SARS-CoV-2 spike protein additionally stained with Alexa Fluor 488 dye (a). Flow cytometry histograms for the uptake of studied Zn_2_GeO_4_:Mn^2+^ NPs by human T-cell leukemia Jurkat cells (b) and pancreatic carcinoma BxPC cells (c).

Due to further investigation of the potential cytotoxic effects of the coated and uncoated Zn_2_GeO_4_:Mn^2+^ NPs, F-actin staining and optical imaging were performed, as shown in Figure 7. This analysis allowed for visualization of the cytoskeletal integrity and cellular morphology following exposure to interaction with Zn_2_GeO_4_:Mn^2+^ NPs: uncoated Zn_2_GeO_4_:Mn^2+^ NPs (positive control) compared to SARS-CoV-2 S1 spike protein functionalized Zn_2_GeO_4_:Mn^2+^ NPs, and finally with untreated BxPC-3 cells (negative control). Cytoskeletal integrity plays a critical role in maintaining the cell shape, mechanical properties, intracellular trafficking, and overall cellular function. The actin cytoskeleton is highly sensitive to external stimuli, including exposure to any type of NPs, and any disruption could indicate potential cytotoxicity or cellular stress. In the case of uncoated Zn_2_GeO_4_:Mn^2+^ NPs, cells exposed to nonfunctionalized PersLNPs exhibited noticeable morphological changes, including cell shrinkage and reduced cell density, suggesting that these native PersLNPs exerted significant stress on the cells. The alterations observed in the cytoskeleton may result from oxidative stress, membrane destabilization, or PersLNPs-induced apoptosis.^43,44^ These findings indicate that uncoated Zn_2_GeO_4_:Mn^2+^ NPs may have inherent cytotoxic properties, possibly as an effect of their direct interactions with the plasma membrane or intracellular organelles. In the case of SARS-CoV-2 S1 spike protein-functionalized Zn_2_GeO_4_:Mn^2+^ NPs, cells maintained their structural integrity, as evidenced by well-preserved actin fibers and normal cellular morphology. No significant cytoskeletal disruptions were detected, indicating that surface functionalization mitigates the cytotoxic effects observed with the noncoated PersLNPs. This suggests that the biofunctionalized PersLNPs surface plays a protective role, reducing direct interactions that may lead to cellular damage or apoptosis. The ability of the SARS-CoV-2 S1 spike protein-functionalized PersLNPs to preserve cytoskeletal integrity and maintain normal cell morphology underscores their biocompatibility, which is crucial for any biomedical applications. The absence of cytoskeletal reorganization or apoptosis-related changes supports the potential safety profile of these engineered nanostructures.^45^ These results were also confirmed through the additional cytotoxicity studies of our systems in MTT assay (BxPc3 cell line) and PrestoBlue assay (Jurkat cells) performed at two different incubation times and on two studied cell lines in a wide range of dilutions (Figure S7). Additionally, an improved cellular response in functionalized PersLNPs-treated cells may be attributed to their enhanced biorecognition and selective uptake via TLR pathways, as we described in the above section (Figure 6). The bioimaging results also shown in Figure 7 indicate the additional potential of the VLP obtained based on Zn_2_GeO_4_:Mn^2+^ NPs in theranostic application. The obtained results validate the effectiveness of the designed Zn_2_GeO_4_:Mn^2+^ NPs system while also demonstrating its potential safety for future *in vivo* applications. We suggest that future studies focus on long-term cellular responses, including potential Zn_2_GeO_4_:Mn^2+^ NPs degradation, immune interactions, and clearance mechanisms. Further validation in animal models will be necessary to assess biodistribution, clearance, and immune system activation to confirm their therapeutic potential.

**Figure 7:**
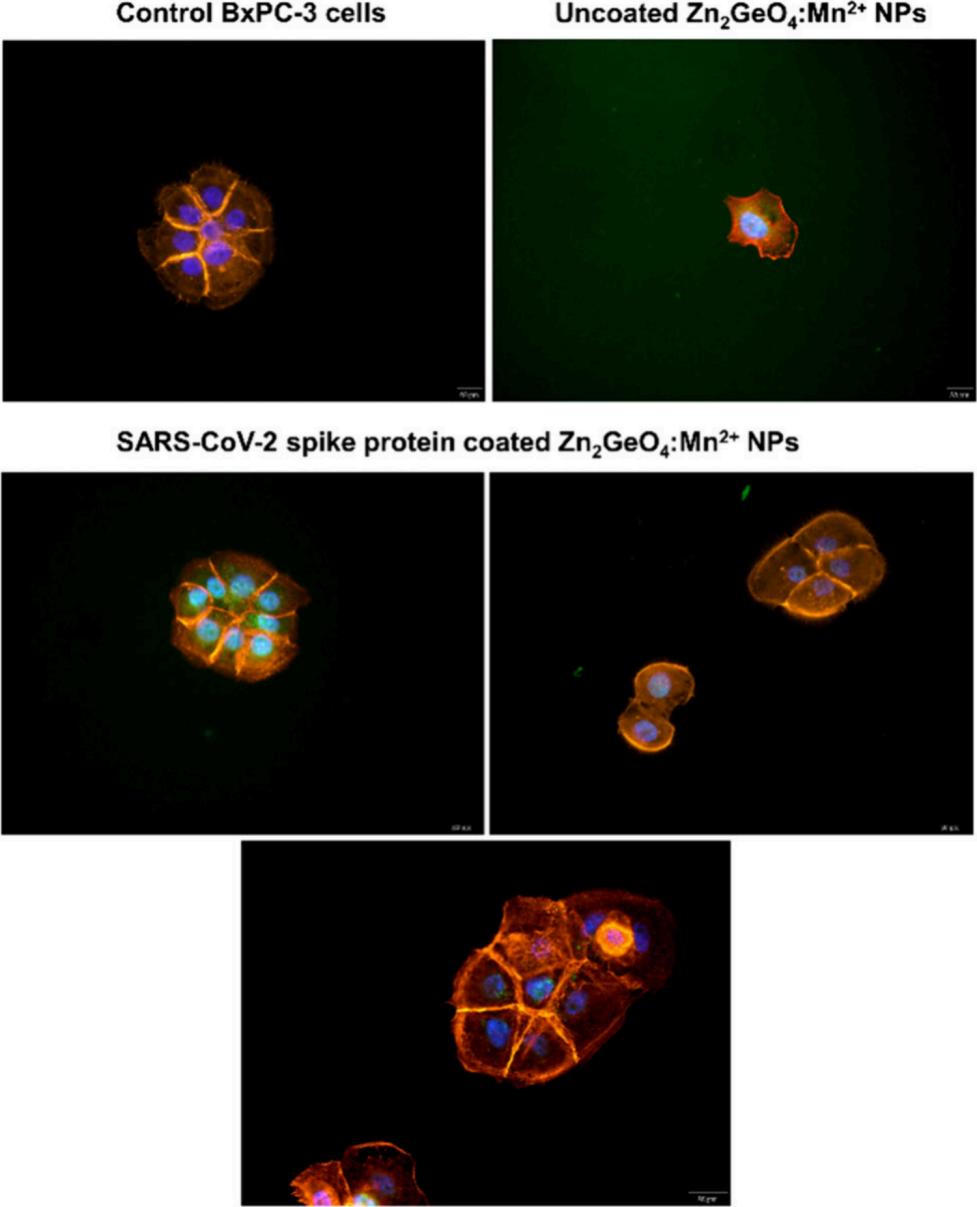
Immunofluorescence F-actin staining performance of pancreatic carcinoma BxPC-3 cells after treatment with the SARS-CoV-2 spike protein modified and uncoated Zn_2_GeO_4_:Mn^2+^ NPs dispersion compared to control untreated cells, where red is F-actin (phalloidin) and blue is nuclei (DAPI). A 400× magnification was used.

## 4 Conclusions

In summary, we rationally designed and developed the strategy of using the virus like particles (VLPs) concept for active targeting of luminescent NPs toward specific cells. The key factor and advantage of the proposed approach relies on the fact that the surface functionalization method used is straightforward, based on basic electrostatic interactions; thus, we believe it can be extrapolated to almost any kind of colloidal NPs. In principle, we have studied the effect of the pH set for the reaction conditions of the morphology, crystal structure, and spectroscopic features (including afterglow signals) of the Mn-doped Zn_2_GeO_4_ NPs. The particles showing the best PersL performance and additionally having a size resembling that of the real virus species were further functionalized with SARS-CoV-2 S1 spike proteins. The efficiency of the surface functionalization was studied by measurements of the surface potential and was also based on TEM imaging. It is of great importance, thus, that the used modification method did not quench the emissive properties of the raw luminescence material, still showing long afterglow signals with a decay time of 60 s after attaching the protein to the surface. Finally, the SARS-CoV-2 S1 spike protein-functionalized Zn_2_GeO_4_:Mn^2+^ NPs were used in biological experiments, showing both decreased cytotoxicity and increased cellular uptake by cells having TLRs. Our results indicate that the concept of VLPs can be used for inorganic NPs toward their more robust biorelated applications.

## Supplementary Material



## Notes

Estimation of the number of SARS-CoV-2 S1 protein molecules attached to the NPs surface, size distribution histograms, additional TEM images, ζ-potential and SDS-PAGE measurement results for functionalized NPs, and cytotoxicity studies (PDF)

## References

[1] Li H., Tursun M., Aihemaiti A., Yan P., Liu X., Abdukayum A. (2025). A Multifunctional Nanoplatform Based on Carbon Nanotubes
Loaded with Persistent Luminescent Nanoparticles for Photocatalysis,
Photothermal Therapy, and Drug Delivery Applications. ACS Appl. Mater. Interfaces.

[2] Feng Y., Liu R., Zhang L., Li Z., Su Y., Lv Y. (2019). Raspberry-Like
Mesoporous Zn1.07Ga2.34Si0.98O6.56:Cr0.01 Nanocarriers for Enhanced
Near-Infrared Afterglow Imaging and Combined Cancer Chemotherapy. ACS Appl. Mater. Interfaces.

[3] Zhu Y., Wen Y., Xie Y., Chen G., Hu S., Wu Y., Jiang L., Viana B., Richard C., Wong K.-L., Jiao J., Wang J., Zou R. (2025). Intelligent Hierarchical
Targeting Near-Infrared Persistent Luminescence Nanosystem for Improved
Nuclear Delivery and Simultaneous Visualization/Therapy of EBV-Associated
Cancer. ACS Appl. Mater. Interfaces.

[4] Xu J., Tanabe S. (2019). Persistent
Luminescence Instead of Phosphorescence:
History, Mechanism, and Perspective. J. Lumin..

[5] Sun X., Song L., Liu N., Shi J., Zhang Y. (2021). Chromium-Doped
Zinc Gallate Near-Infrared Persistent Luminescence Nanoparticles in
Autofluorescence-Free Biosensing and Bioimaging: A Review. ACS Appl. Nano Mater..

[6] Wu S., Li Y., Ding W., Xu L., Ma Y., Zhang L. (2020). Recent Advances
of Persistent Luminescence Nanoparticles in Bioapplications. Nanomicro Lett..

[7] Suárez P. L., García-Cortés M., Fernández-Argüelles M. T., Encinar J. R., Valledor M., Ferrero F. J., Campo J. C., Costa-Fernández J. M. (2019). Functionalized
Phosphorescent Nanoparticles
in (Bio)­Chemical Sensing and Imaging - A Review. Anal. Chim. Acta.

[8] Mitchell M. J., Billingsley M. M., Haley R. M., Wechsler M. E., Peppas N. A., Langer R. (2021). Engineering Precision Nanoparticles
for Drug Delivery. Nat. Rev. Drug Discov.

[9] Saker R., Regdon G., Sovány T. (2024). Pharmacokinetics and Toxicity of
Inorganic Nanoparticles and the Physicochemical Properties/Factors
Affecting Them. J. Drug Deliv Sci. Technol..

[10] Shi L., Shao J., Jing X., Zheng W., Liu H., Zhao Y. (2020). Autoluminescence-Free
Dual Tumor Marker Biosensing by Persistent
Luminescence Nanostructures. ACS Sustain Chem.
Eng..

[11] Wang J., Ma Q., Zheng W., Liu H., Yin C., Wang F., Chen X., Yuan Q., Tan W. (2017). One-Dimensional
Luminous
Nanorods Featuring Tunable Persistent Luminescence for Autofluorescence-Free
Biosensing. ACS Nano.

[12] Wang J., Ma Q., Liu H., Wang Y., Shen H., Hu X., Ma C., Yuan Q., Tan W. (2017). Time-Gated Imaging of Latent Fingerprints
and Specific Visualization of Protein Secretions via Molecular Recognition. Anal. Chem..

[13] Chen S., Cai G., Gong X., Wang L., Cai C., Gong H. (2022). Non-Autofluorescence
Detection of H5N1 Virus Using Photochemical Aptamer Sensors Based
on Persistent Luminescent Nanoparticles. ACS
Appl. Mater. Interfaces.

[14] Calderón-Olvera R. M., Arroyo E., Jankelow A. M., Bashir R., Valera E., Ocaña M., Becerro A. I. (2023). Persistent Luminescence Zn2GeO4:Mn2+
Nanoparticles Functionalized with Polyacrylic Acid: One-Pot Synthesis
and Biosensing Applications. ACS Appl. Mater.
Interfaces.

[15] Bhattacharjee K., Prasad B. L. V. (2023). Surface Functionalization of Inorganic Nanoparticles
with Ligands: A Necessary Step for Their Utility. Chem. Soc. Rev..

[16] Li L., Jiang X., Gao J. (2022). Characterization
and Biomedical Application
Opportunities of the Nanoparticle’s Protein Corona. Adv. Mater. Interfaces.

[17] Mahmoudi M., Bertrand N., Zope H., Farokhzad O. C. (2016). Emerging
Understanding of the Protein Corona at the Nano-Bio Interfaces. Nano Today.

[18] Caracciolo G. (2024). Artificial
Protein Coronas: Directing Nanoparticles to Targets. Trends Pharmacol. Sci..

[19] Malhotra K., Kumar B., Piunno P. A. E., Krull U. J. (2024). Cellular Uptake
of Upconversion Nanoparticles Based on Surface Polymer Coatings and
Protein Corona. ACS Appl. Mater. Interfaces.

[20] Zhao T., Ren M., Shi J., Wang H., Bai J., Du W., Xiang B. (2024). Engineering the Protein Corona: Strategies, Effects, and Future Directions
in Nanoparticle Therapeutics. Biomedicine &
Pharmacotherapy.

[21] Nooraei, S. ; Bahrulolum, H. ; Hoseini, Z. S. ; Katalani, C. ; Hajizade, A. ; Easton, A. J. ; Ahmadian, G. Virus-like Particles: Preparation, Immunogenicity and Their Roles as Nanovaccines and Drug Nanocarriers. J. Nanobiotechnol. 2021, 19 (1). 10.1186/s12951-021-00806-7. PMC7905985 33632278

[22] Sun X., Lian Y., Tian T., Cui Z. (2024). Advancements in Functional
Nanomaterials Inspired by Viral Particles. Small.

[23] Khanmohammadi S., Rezaei N. (2021). Role of Toll-like Receptors
in the Pathogenesis of
COVID-19. J. Med. Virol.

[24] Vaz J., Andersson R. (2014). Intervention
on Toll-like Receptors in Pancreatic Cancer. World J. Gastroenterol.

[25] Ferreira P. S., Gerbelli B. B., Cantero J., Iribarne F., de Castro-Kochi A. C.
H., Kochi L. T., Castro F. L., Alves W. A. (2025). Influence of Cholesterol
on the Insertion and Interaction of SARS-CoV-2 Proteins with Lipid
Membranes. ACS Appl. Bio Mater..

[26] Neblea I. E., Iordache T. V., Sarbu A., Chiriac A. L., Gavrila A. M., Trica B., Biru I. E., Caras I., Teodorescu M., Perrin F. X., Zaharia A. (2025). Biomimetic
Molecularly Imprinted
Nanogels for the Recognition of Spike Glycoproteins. ACS Appl. Bio Mater..

[27] Maity S., Acharya A. (2024). Many Roles of Carbohydrates:
A Computational Spotlight
on the Coronavirus S Protein Binding. ACS Appl.
Bio Mater..

[28] Andrzejewska W., Peplińska B., Litowczenko J., Obstarczyk P., Olesiak-Bańska J., Jurga S., Lewandowski M. (2023). SARS-CoV-2
Virus-like Particles with Plasmonic Au Cores and S1-Spike Protein
Coronas. ACS Synth. Biol..

[29] Bauer, D. ; Ashton, T. E. ; Groves, A. R. ; Dey, A. ; Krishnamurthy, S. ; Matsumi, N. ; Darr, J. A. Continuous Hydrothermal Synthesis of Metal Germanates (M_2_GeO_4_; M = Co, Mn, Zn) for High-Capacity Negative Electrodes in Li-Ion Batteries. Energy Technol. 2020, 8 (1). 10.1002/ente.201900692.

[30] Wan M., Wang Y., Wang X., Zhao H., Li H., Wang C. (2014). Long Afterglow Properties
of Eu2+/Mn2+ Doped Zn 2GeO4. J. Lumin..

[31] Dias M. P., Batista M. S., Pimentel A., Fortunato E., Martins R., Costa F. M., Pereira S. O., Rodrigues J., Monteiro T. (2025). Revealing the Nature of the Zn_2_GeO_4_ Bluish-White Emission in Microwave-Assisted
Hydrothermal Synthesized
Nanorods. Mater. Chem. Phys..

[32] Xue J., Li F., Liu F., Noh H. M., Lee B. R., Choi B. C., Park S. H., Jeong J. H., Du P. (2022). Designing Ultra-Highly
Efficient Mn^2+^-Activated Zn_2_GeO_4_ Green-Emitting
Persistent Phosphors toward Versatile Applications. Mater. Today Chem..

[33] Sun X. Y., He Z., Gu X. (2018). Persistent Luminescence of Zn2GeO4:Mn2+/Pr3+ Phosphors. Journal of Materials Science: Materials in Electronics.

[34] Huang, K. ; Dou, X. ; Zhang, Y. ; Gao, X. ; Lin, J. ; Qu, J. ; Li, Y. ; Huang, P. ; Han, G. Enhancing Light and X-Ray Charging in Persistent Luminescence Nanocrystals for Orthogonal Afterglow Anti-Counterfeiting. Adv. Funct Mater. 2021, 31 (22). 10.1002/adfm.202009920.

[35] Castaing, V. ; Arroyo, E. ; Becerro, A. I. ; Ocaña, M. ; Lozano, G. ; Míguez, H. Persistent Luminescent Nanoparticles: Challenges and Opportunities for a Shimmering Future. J. Appl. Phys. 2021, 130 (8). 10.1063/5.0053283.

[36] Du J., De Clercq O. Q., Poelman D. (2019). Temperature Dependent Persistent
Luminescence: Evaluating the Optimum Working Temperature. Sci. Rep.

[37] Gao F., Pang Q., Gao D., Jia C., Xin H., Pan Y., Wang Y., Yun S. (2023). Mn2+-Activated Photostimulable Persistent
Nanophosphors by Pr3+ Codoping for Rewritable Information Storage. ACS Appl. Nano Mater..

[38] Singh, T. B. ; Chanu, L. P. ; Gartia, R. K. Determination of Electron Trapping Parameters: A Revisit to Urbach’s Formula. Journal of Physics: Conference Series; Institute of Physics Publishing, 2019; Vol. 1330. 10.1088/1742-6596/1330/1/012011.

[39] Pereyda-Pierre C., Meléndrez R., García R., Pedroza-Montero M., Barboza-Flores M. (2011). Persistent
Luminescence and Thermoluminescence of UV/VIS
-Irradiated SrAl2O4: Eu2+, Dy3+ Phosphor. Radiat.
Meas..

[40] Laue, M. ; Kauter, A. ; Hoffmann, T. ; Möller, L. ; Michel, J. ; Nitsche, A. Morphometry of SARS-CoV and SARS-CoV-2 Particles in Ultrathin Plastic Sections of Infected Vero Cell Cultures. Sci. Rep. 2021, 11 (1). 10.1038/s41598-021-82852-7. PMC7876034 33568700

[41] Totura, A. L. ; Whitmore, A. ; Agnihothram, S. ; Schafer, A. ; Katze, M. G. ; Heise, M. T. ; Baric, R. S. Toll-Like Receptor 3 Signaling via TRIF Contributes to a Protective Innate Immune Response to Severe Acute Respiratory Syndrome Coronavirus Infection. mBio 2015, 6 (3). 10.1128/mBio.00638-15. PMC4447251 26015500

[42] Rakoff-Nahoum S., Medzhitov R. (2009). Toll-like
Receptors and Cancer. Nat. Rev. Cancer.

[43] Ziegler U., Groscurth P. (2004). Morphological
Features of Cell Death. Physiology.

[44] Yurinskaya V., Goryachaya T., Guzhova I., Moshkov A., Rozanov Y., Sakuta G., Shirokova A., Shumilina E., Vassilieva I., Lang F., Vereninov A. (2005). Potassium
and Sodium Balance in U937 Cells During Apoptosis With and Without
Cell Shrinkage. Cellular Physiology and Biochemistry.

[45] Cummings B. S., Schnellmann R. G. (2021). Measurement of Cell Death in Mammalian Cells. Curr. Protoc.

