# Supplementary material for: Persistent Green Luminescence in Nanoparticles Functionalized with SARS-CoV‑2 Spike Proteins: Virus-Like Particles Showing Active Targeting toward Selected Cells
Source: ACS Appl Bio Mater. 2025 Oct 26;8(11):10038–49. doi: 10.1021/acsabm.5c01355 (PMC12628321; doi:10.1021/acsabm.5c01355)
Supplement: Supplementary file 1 [file mt5c01355_si_001.pdf]

Supporting Information

for

Persistent green luminescence in nanoparticles functionalized with SARS-CoV-2 spike proteins – virus like particles showing active targeting towards selected cells

Piotr Kuich[1], Urszula Bazylińska[2], Julita Kulbacka[3,4], Vitalii Boiko[5,6], Dariusz Hreniak[5], Michał Jewgiński[7], Marcin Nyk[1] and Dominika Wawrzyńczyk[1]*

[1] *Institute of Advanced Materials, Faculty of Chemistry, Wroclaw University of Science and Technology, Wybrzeze Wyspianskiego 27, 50-370, Wroclaw, Poland*

[2] *Department of Physical and Quantum Chemistry, Faculty of Chemistry, Wroclaw University of Science and Technology, Wybrzeze Wyspianskiego 27, 50-370, Wroclaw, Poland.*

[3] *Department of Molecular and Cellular Biology, Faculty of Pharmacy, Wroclaw Medical University, Borowska 211 A, 50-556 Wroclaw, Poland*

[4] *Department of Immunology and Bioelectrochemistry, State Research Institute Centre for Innovative Medicine, LT-08406 Vilnius, Lithuania*

[5] *Division of Optical Spectroscopy, Institute of Low Temperature and Structure Research Polish Academy of Sciences, Okolna 2, 50-422 Wroclaw, Poland*

[6] *Department of Physics of Biological Systems, Institute of Physics, National Academy of Sciences of Ukraine, Prospekt Nauky 46, UA-03028, Kyiv, Ukraine*

[7] *Department of Bioorganic Chemistry, Faculty of Chemistry, Wroclaw University of Science and Technology, Wybrzeze Wyspianskiego 27, 50-370, Wroclaw, Poland*

** Corresponding author: dominika.wawrzynczyk@pwr.edu.pl*

The number of SARS-CoV-2 S1 protein molecules attached to the nanoparticles surface was roughly estimated based on measurements of protein absorption calibration curve (Figure S1). 4 concentrations of the S1 protein have been prepared, i.e. 5, 10, 15 and 20 μg/mL, of the total volume 0.1 mL each, and for such prepared solutions, UV-Vis spectra have been measured and calibration curve obtained. Next, the nanoparticles functionalized with a SARS-CoV-2 S1 protein concentration in the solution of 15 and 20 μg/mL are centrifugated (5 000 rpm, 5 min), and the amount of the protein (unbounded) left in the supernatant is validated based on the absorption measurements and comparing to the calibration curve. As the result of this experiment S1 protein concentration in the supernatant was roughly estimated to be 9.7 and 6.8 μg/mL for the samples functionalized with S1 protein solution of concentration of 20 and 15 μg/mL, respectively. From the above it seems that the concentration of SARS-CoV-2 S1 protein equal to ~10 μg/mL used for functionalization should fully saturate the NPs surface. Thus, this

value is taken for further calculations. Taking into account volume of the solution and the molar mass of the S1 protein, the number of moles of proteins attached to the nanoparticles surface could be estimated at the level of 10^{-11}. Next, taking into account the Avogadro's number and knowing the amount of the nanoparticles used during the functionalization process, the amount of SARS-CoV-2 S1 protein per single nanoparticle should be at the level of 10^{4}.

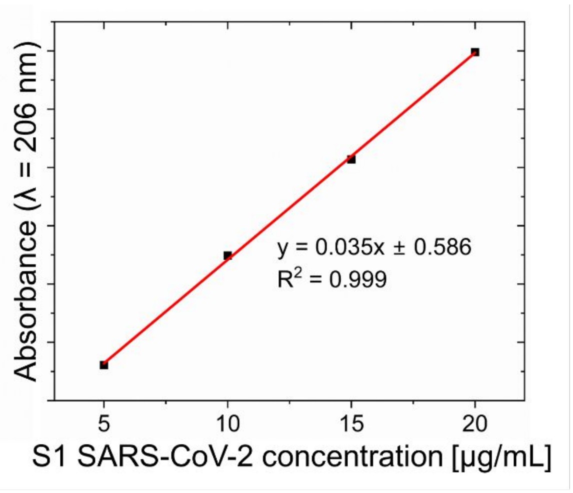

Figure S1. Calibration curve obtained based on the value of absorbance measured at λ = 206 nm in dependence of SARS-CoV-2 S1 protein concentration in the solution.

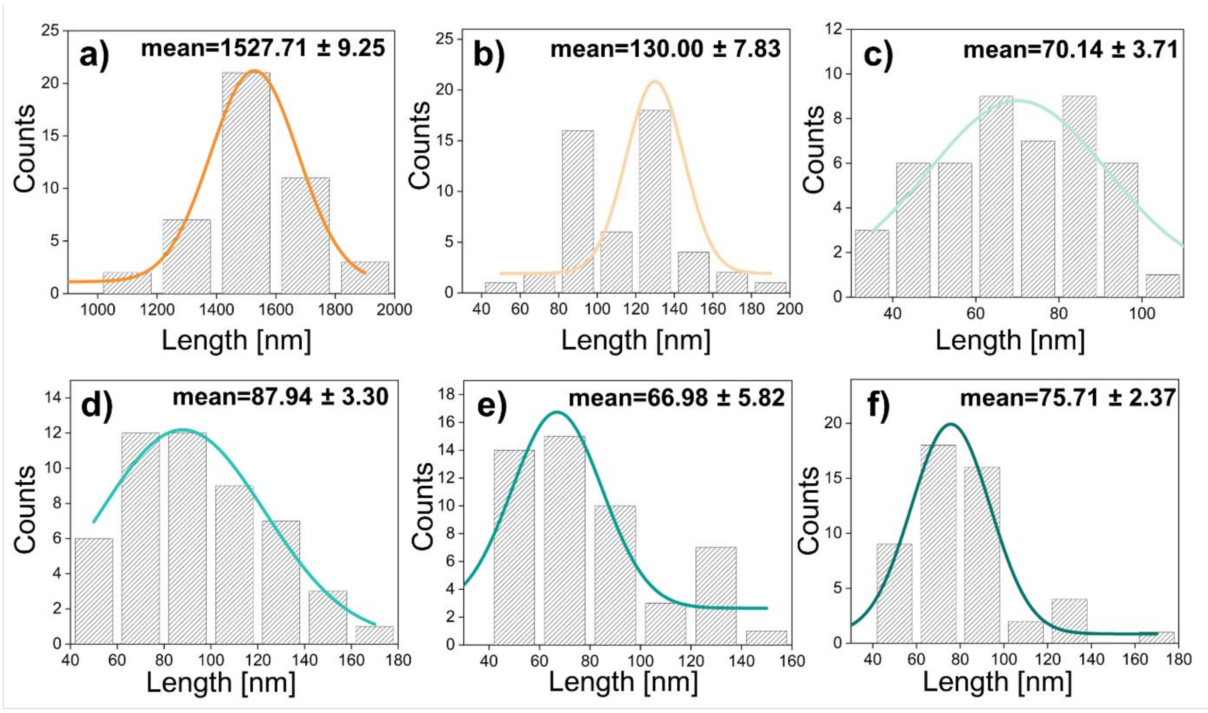

Figure S2. Size distribution histograms of length for materials obtained in different pH values, set during the synthesis (a)-(f), i.e. 6.0; 7.0; 7.5; 8.0; 8.5 and 9.5, respectively

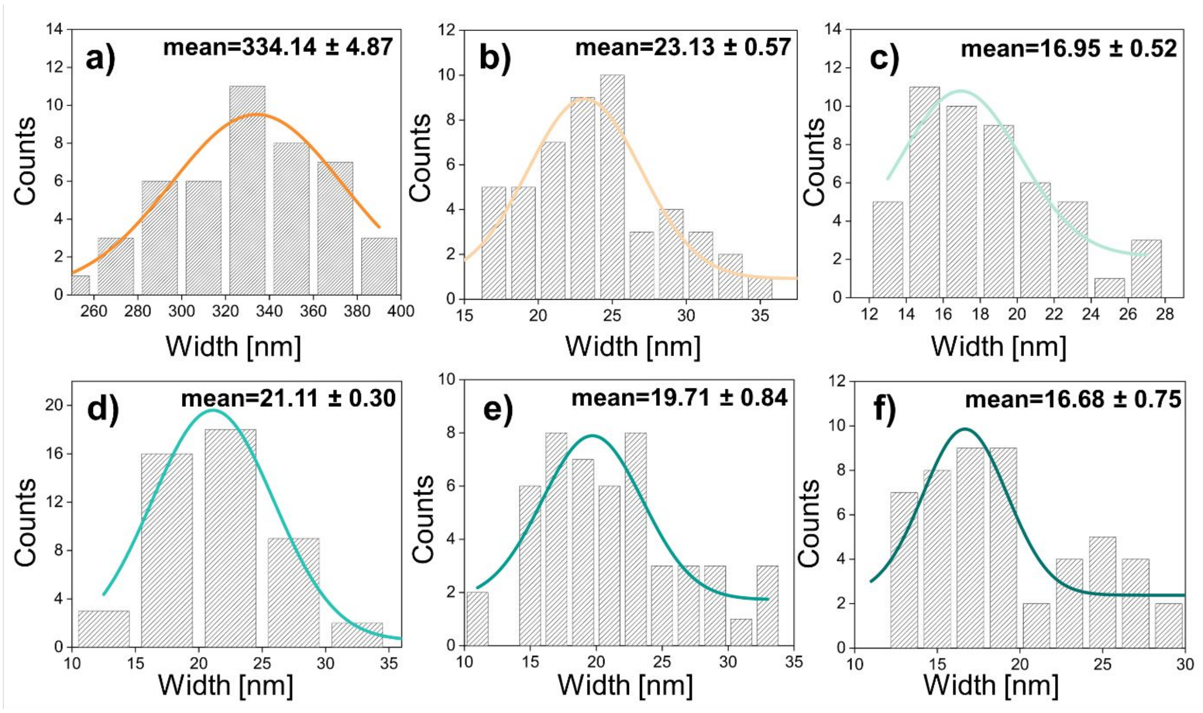

Figure S3. Size distribution histograms of widths (a)-(f) for materials obtained in different pH values, set during the synthesis, i.e. 6.0; 7.0; 7.5; 8.0; 8.5 and 9.5, respectively.

Table S1. The main parameters characterizing $Zn_2GeO_4:Mn^{2+}$ NPs synthesized at different reaction pH.

Synthesis pH	Length [nm]	Width [nm]	PersL decay $\tau_{1/2}$ [s]	Trap depths [eV]
6.0	1527.71 ± 9.25	334.14 ± 4.87	87.52 ± 1.46	0.68
7.0	130.00 ± 7.83	23.13 ± 0.57	86.26 ± 1.17	0.69
7.5	70.14 ± 3.71	16.95 ± 0.52	75.61 ± 0.90	0.68
8.0	87.94 ± 3.30	21.11 ± 0.30	69.29 ± 0.78	0.67
8.5	66.98 ± 5.82	19.71 ± 0.84	67.76 ± 0.74	0.67
9.5	75.71 ± 2.37	16.68 ± 0.75	70.60 ± 0.78	0.67

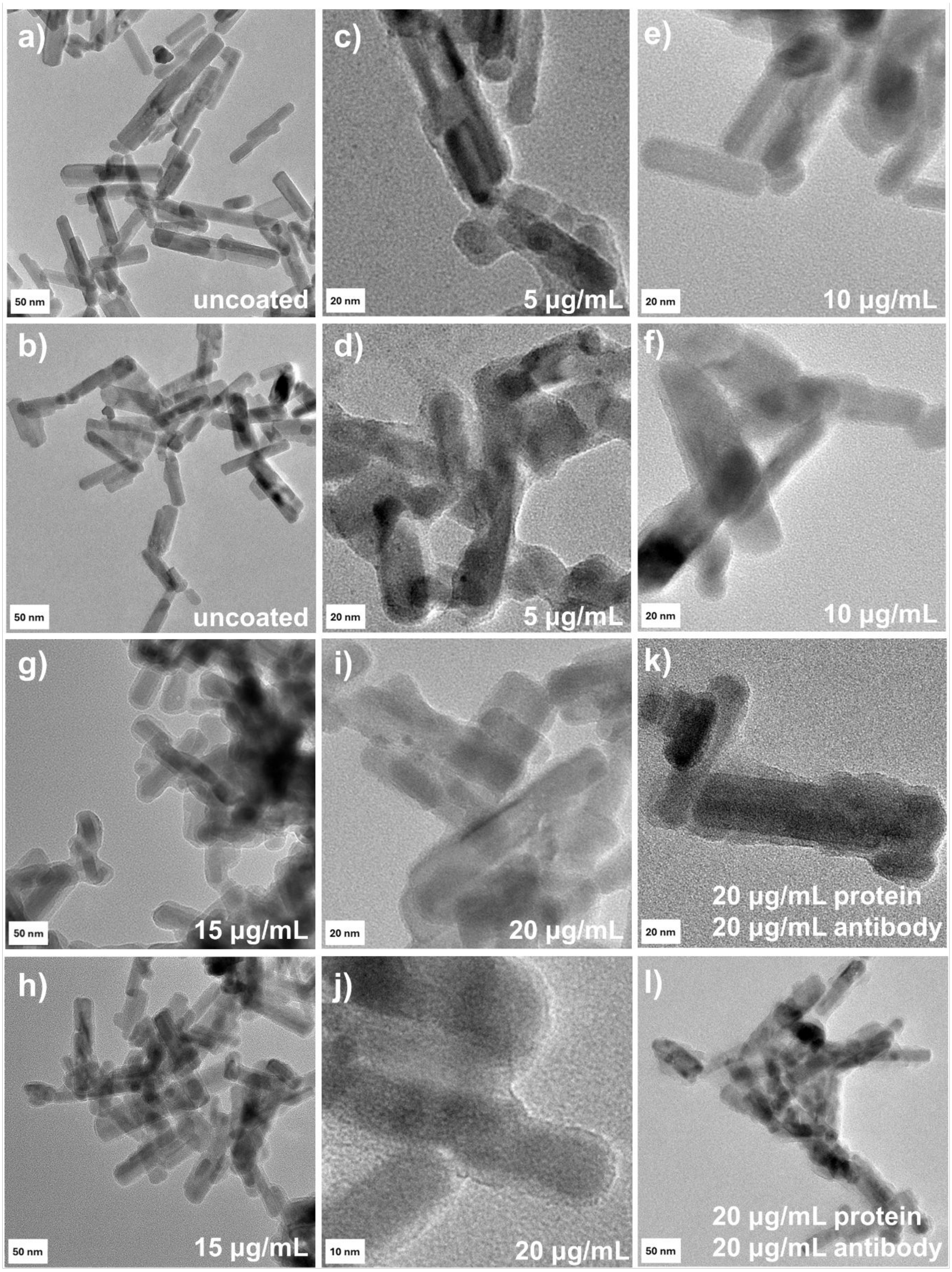

Figure S4. TEM images of Zn_2GeO_4:Mn NPs obtained in pH 9.5, as NPs unmodified with S1 SARS-CoV-2 (a)-(b), modified with S1 SARS-CoV-2 protein of: 5 µg/mL (c)-(d), 10 µg/mL (e)-(f), 15 µg/mL (g)-(h), 20 µg/mL (i)-(j) and 20 µg/mL with addition of 20 µg/mL specific antibodies (k)-(l).

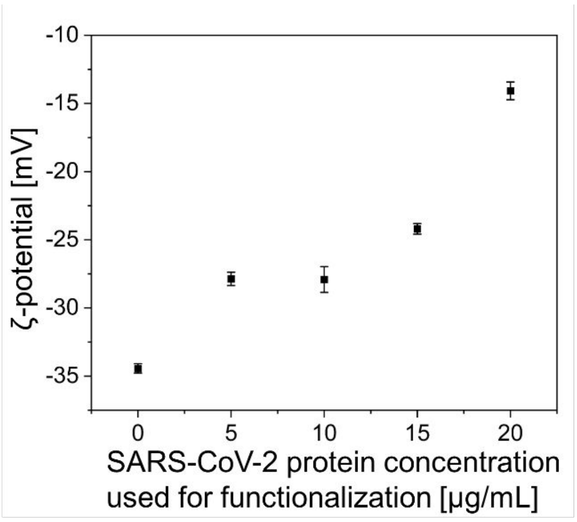

Figure S5. Measured values of ζ-potential for reference sample, i.e. Zn_2GeO_4:Mn NPs without SARS-CoV-2 S1 protein used for surface functionalization - 0 µg/mL, as well as for all SARS-CoV-2 S1 protein concentrations - 5, 10, 15 and 20 µg/mL, respectively.

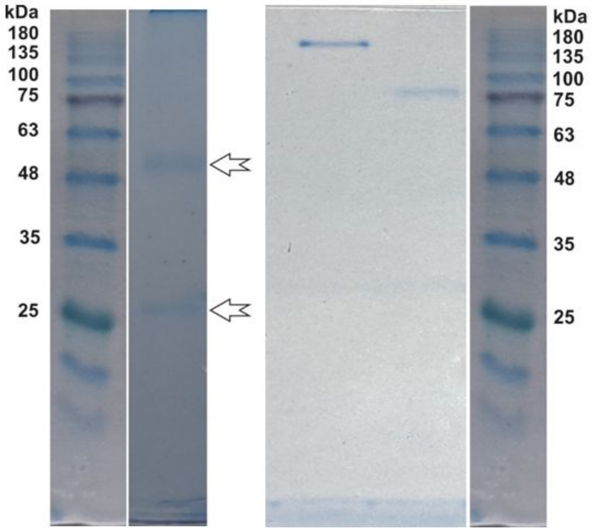

Figure S6. Results of SDS-PAGE of SARS-Cov-2-S1 antibodies (left panel) and nondenatirating PAGE (right panel) of SARS-Cov-2-S1 antibodies (left line) and functionalized Zn_2GeO_4:Mn NPs (middle line). Both panels include Perfect Tricolor Protein Ladder (EURx, cat no. E3210)

In the nondenaturing PAGE the SARS-Cov-2-S1 antibody migrated with an apparent molecular mass of 180kDa, whereas in the SDS-PAGE with the presence of DTT, two molecular masses appear corresponding to approximately 30kDa and 60kDa, what is in agreement with the characteristic provided by the producer. In the same condition on nondenaturating PAGE functionalized Zn_2GeO_4:Mn NPs + SARS-Cov-2-S1 protein conjugates migrated with an apparent molecular mass around 90kDa. The obtained results are close to the molecular mass of isolated SARS-Cov-2-S1 protein, suggesting that analyzed conjugates of protein with

Zn_2GeO_4:Mn NPs probably is unstable under the experimental condition, leading to their decomposition. This observation seems to be confirmed by the fact that attempts to visualize the analyzed samples with UV light (not shown), after the completed electrophoresis did not reveal the presence of nanoparticles.

Cell viability was determined by MTT assay [Szlasa WK, Sauer NJ, Karwacki J, et al. Avelumab reduces STAT3 expression with effects on IL-17RA and CD15. Dent Med Probl. 2024;61(5):713–720. doi:10.17219/dmp/176374] for BcPC-3 cells, and PrestoBlue® [Weżgowiec J, Małysa A, Szlasa W, Kulbacka J, Chwiłkowska A, Ziętek M, Więckiewicz M. Biocompatibility of 3D-printed vs. thermoformed and heat-cured intraoral appliances. Front Bioeng Biotechnol. 2024 Oct 29;12:1453888] for Jurkat-T cells. Cells were seeded on 96-well plates (T hermo Fisher) at a count of 5 × 10^3 cells per well Cells were exposed to NPs and NPs functionalized by SarsCov-2 protein in the following rations: 1:500, 1:200, 1:100, 1:50, 1:20 for 24 and 48 h to assess the viability changes over time. The absorbance of each well was measured at 560 nm using the multiplate reader (Glomax, Promega, GmbH, Germany). The results were expressed as the percentage of viable cells relative to untreated control cells.

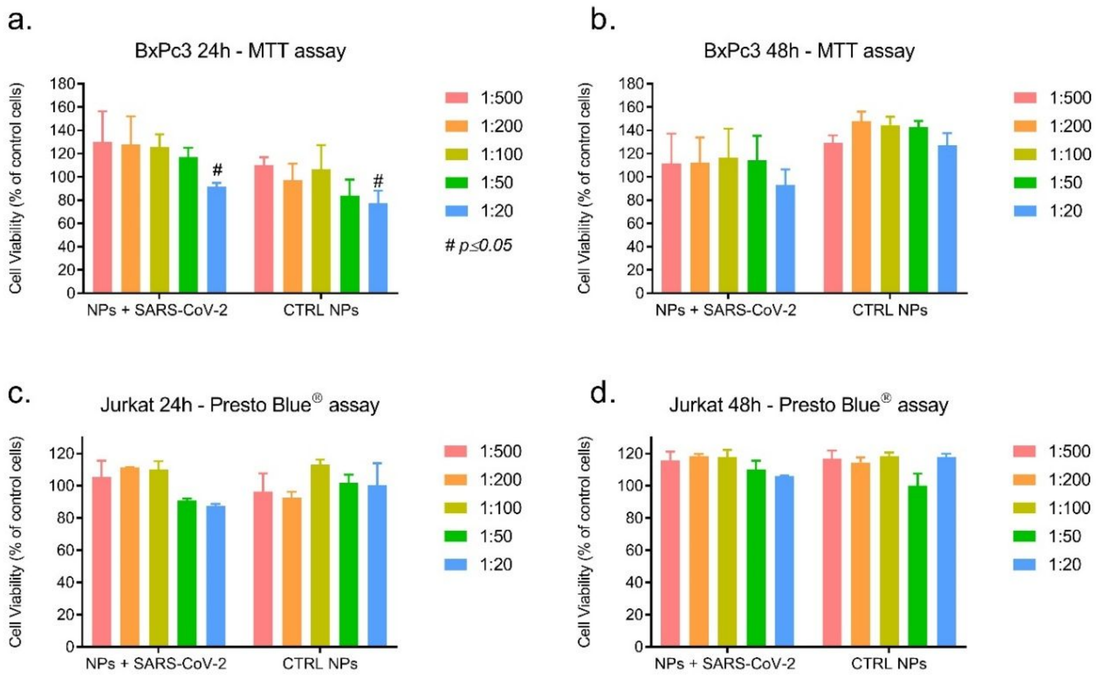

Figure S7. The evaluation of cytotoxicity of functionalized nanoparticles in pancreatic cancer cells BxPc3 after 24h (a) and 48h (b) determined by MTT assay; and in leukemia Jurkat cells after 24h (c) and 48h (d) determined by PrestoBlue assay®.
